# Implications of early respiratory support strategies on disease progression in critical COVID-19: a matched subanalysis of the prospective RISC-19-ICU cohort

**DOI:** 10.1186/s13054-021-03580-y

**Published:** 2021-05-25

**Authors:** Pedro D. Wendel Garcia, Hernán Aguirre-Bermeo, Philipp K. Buehler, Mario Alfaro-Farias, Bernd Yuen, Sascha David, Thomas Tschoellitsch, Tobias Wengenmayer, Anita Korsos, Alberto Fogagnolo, Gian-Reto Kleger, Maddalena A. Wu, Riccardo Colombo, Fabrizio Turrini, Antonella Potalivo, Emanuele Rezoagli, Raquel Rodríguez-García, Pedro Castro, Arantxa Lander-Azcona, Maria C. Martín-Delgado, Herminia Lozano-Gómez, Rolf Ensner, Marc P. Michot, Nadine Gehring, Peter Schott, Martin Siegemund, Lukas Merki, Jan Wiegand, Marie M. Jeitziner, Marcus Laube, Petra Salomon, Frank Hillgaertner, Alexander Dullenkopf, Hatem Ksouri, Sara Cereghetti, Serge Grazioli, Christian Bürkle, Julien Marrel, Isabelle Fleisch, Marie-Helene Perez, Anja Baltussen Weber, Samuele Ceruti, Katharina Marquardt, Tobias Hübner, Hermann Redecker, Michael Studhalter, Michael Stephan, Daniela Selz, Urs Pietsch, Anette Ristic, Antje Heise, Friederike Meyer zu Bentrup, Marilene Franchitti Laurent, Patricia Fodor, Tomislav Gaspert, Christoph Haberthuer, Elif Colak, Dorothea M. Heuberger, Thierry Fumeaux, Jonathan Montomoli, Philippe Guerci, Reto A. Schuepbach, Matthias P. Hilty, Ferran Roche-Campo, RISC-19-ICU Investigators, Pedro D. Wendel Garcia, Hernán Aguirre-Bermeo, Philipp K. Buehler, Mario Alfaro-Farias, Bernd Yuen, Sascha David, Thomas Tschoellitsch, Tobias Wengenmayer, Anita Korsos, Alberto Fogagnolo, Gian-Reto Kleger, Maddalena A. Wu, Riccardo Colombo, Fabrizio Turrini, Antonella Potalivo, Emanuele Rezoagli, Raquel Rodríguez-García, Pedro Castro, Arantxa Lander-Azcona, Maria C. Martín-Delgado, Herminia Lozano-Gómez, Rolf Ensner, Marc P. Michot, Nadine Gehring, Peter Schott, Martin Siegemund, Lukas Merki, Jan Wiegand, Marie M. Jeitziner, Marcus Laube, Petra Salomon, Frank Hillgaertner, Alexander Dullenkopf, Hatem Ksouri, Sara Cereghetti, Serge Grazioli, Christian Bürkle, Julien Marrel, Isabelle Fleisch, Marie-Helene Perez, Anja Baltussen Weber, Samuele Ceruti, Katharina Marquardt, Tobias Hübner, Hermann Redecker, Michael Studhalter, Michael Stephan, Daniela Selz, Urs Pietsch, Anette Ristic, Antje Heise, Friederike Meyer zu Bentrup, Marilene Franchitti Laurent, Patricia Fodor, Tomislav Gaspert, Christoph Haberthuer, Elif Colak, Dorothea M. Heuberger, Thierry Fumeaux, Jonathan Montomoli, Philippe Guerci, Reto A. Schuepbach, Matthias P. Hilty, Ferran Roche-Campo, Angela Algaba-Calderon, Janina Apolo, Theodoros Aslanidis, Barna Babik, Filippo Boroli, Jan Brem, Mirko Brenni, Silvio D. Brugger, Giovanni Camen, Emanuele Catena, Roberto Ceriani, Ivan Chau, Andreas Christ, Chiara Cogliati, Pablo Concha, Gauthier Delahaye, Iris Drvaric, Jesús Escós-Orta, Silvia Fabbri, Francesca Facondini, Miodrag Filipovic, Jorge Gámez-Zapata, Peter Gerecke, Diederik Gommers, Thomas Hillermann, Can Ince, Beatrice Jenni-Moser, Marija Jovic, Geoffrey Jurkolow, Alexander Klarer, Adriana Lambert, Jean-Christophe Laurent, Jerome Lavanchy, Barbara Lienhardt-Nobbe, Pascal Locher, Marie-Reine Losser, Roger F. Lussman, Aurora Magliocca, Antoni Margarit, Alberto Martínez, Romano Mauri, Eric Mayor-Vázquez, Jens Meier, Mallory Moret-Bochatay, Martina Murrone, Didier Naon, Thomas Neff, Emmanuel Novy, Lina Petersen, Jerome Pugin, Anne-Sylvie Ramelet, Jonathan Rilinger, Peter C. Rimensberger, Michael Sepulcri, Karim Shaikh, Marianne Sieber, Maria Sole Simonini, Savino Spadaro, Govind Oliver Sridharan, Klaus Stahl, Dawid L. Staudacher, Xiana Taboada-Fraga, Adrian Tellez, Severin Urech, Giovanni Vitale, Gerardo Vizmanos-Lamotte, Tobias Welte, Begoña Zalba-Etayo, Nuria Zellweger

**Affiliations:** 1 Institute of Intensive Care Medicine, University Hospital of Zurich, Zurich, Switzerland; 2 The RISC-19-ICU Registry Board, University of Zurich, Zurich, Switzerland; 3 Unidad de Cuidados Intensivos, Hospital Vicente Corral Moscoso, Cuenca, Ecuador; 4 Unidad de Cuidados Intensivos, Hospital Nostra Senyora de Meritxell, Escaldes-Engordany, Andorra; 5 Interdisziplinaere Intensivstation, Spital Buelach, Buelach, Switzerland; 6 Department of Nephrology and Hypertension, Medical School Hannover, Hannover, Germany; 7 Department of Anesthesiology and Critical Care Medicine, Kepler University Hospital GmbH and Johannes Kepler University, Linz, Austria; 8 Department of Medicine III - Interdisciplinary Medical Intensive Care, Medical Center University of Freiburg, Freiburg, Germany; 9 Departement of Anaethesiology and Intensive Care, University of Szeged, Szeged, Hungary; 10 Anesthesia and Intensive Care, Azienda Ospedaliero-Universitaria di Ferrara, Cona, Italy; 11 Medizinische Intensivstation, Kantonsspital St. Gallen, St. Gallen, Switzerland; 12 Department of Internal Medicine, ASST Fatebenefratelli Sacco - “Luigi Sacco” Hospital, Milan, Italy; 13 Division of Anesthesia and Intensive Care, ASST Fatebenefratelli Sacco - “Luigi Sacco” Hospital, Milan, Italy; 14 Internal Medicine, Azienda Ospedaliera Universitaria di Modena, Modena, Italy; 15 UOC Anestesia e Rianimazione, Ospedale Infermi, Rimini, Italy; 16 Department of Anesthesia and Intensive Care Medicine, Policlinico San Marco, Gruppo Ospedaliero San Donato, Bergamo, Italy; 17 Servicio de Medicina intensiva, Complejo Hospitalario Universitario A Coruña, A Coruña, Spain; 18 Medical Intensive Care Unit, Hospital Clínic de Barcelona, Barcelona, Spain; 19 Servicio de Medicina Intensiva, Hospital General San Jorge, Huesca, Spain; 20 Servicio de Medicina Intensiva, Hospital Universitario de Torrejón, Madrid, Spain; 21 Unidad de Cuidados Intensivos, Hospital Clínico Universitario Lozano Blesa, Zaragoza, Spain; 22 Klinik für Operative Intensivmedizin, Kantonsspital Aarau, Aarau, Switzerland; 23 Medizinische Intensivstation, Kantonsspital Aarau, Aarau, Switzerland; 24 Intensivstation, Kantonsspital Schaffhausen, Schaffhausen, Switzerland; 25 Institut fuer Anesthaesie und Intensivmedizin, Zuger Kantonsspital AG, Baar, Switzerland; 26 Department Intensivmedizin, Universitaetsspital Basel, Basel, Switzerland; 27 Intensivmedizin, St. Claraspital, Basel, Switzerland; 28 Interdisziplinaere Intensivmedizin, Lindenhofspital, Bern, Switzerland; 29 Department of Intensive Care Medicine, University Hospital Bern, Inselspital, Bern, Switzerland; 30 Department Intensive Care Medicine, Spitalzentrum Biel, Biel, Switzerland; 31 Intensivstation, Regionalspital Emmental AG, Burgdorf, Switzerland; 32 Intensivmedizin, Kantonsspital Graubuenden, Chur, Switzerland; 33 Institut fuer Anaesthesie und Intensivmedizin, Spital Thurgau, Frauenfeld, Switzerland; 34 Soins Intensifs, Hopital cantonal de Fribourg, Fribourg, Switzerland; 35 Division of Intensive Care, University Hospitals of Geneva, Geneva, Switzerland; 36 Division of Neonatal and Pediatric Intensive Care, University Hospitals of Geneva, Geneva, Switzerland; 37 Intensivstation, Spital Grabs, Grabs, Switzerland; 38 Institut für Anaesthesiologie Intensivmedizin & Rettungsmedizin, See-Spital Horgen & Kilchberg, Horgen, Switzerland; 39 Soins Intensifs, Hirslanden Clinique Cecil, Lausanne, Switzerland; 40 Pediatric Intensive Care Unit, University Hospital Lausanne, Lausanne, Switzerland; 41 Anaesthesie und Intensivmedizin, Kantonsspital Baselland, Liestal, Switzerland; 42 Dipartimento Area Critica, Clinica Luganese Moncucco, Lugano, Switzerland; 43 Interdisziplinaere Intensivstation, Spital Maennedorf AG, Maennedorf, Switzerland; 44 Institut fuer Anaesthesie und Intensivmedizin, Spital Thurgau, Muensterlingen, Switzerland; 45 Intensivmedizin, Schweizer Paraplegikerzentrum Nottwil, Nottwil, Switzerland; 46 Intensivmedizin & Intermediate Care, Kantonsspital Olten, Olten, Switzerland; 47 Intensivmedizin, Spital Oberengadin, Samedan, Switzerland; 48 Anaesthesie Intensivmedizin Schmerzmedizin, Spital Schwyz, Schwyz, Switzerland; 49 Departement of Anesthesiology and Intensive Care Medicine, Kantonsspital St. Gallen, St. Gallen, Switzerland; 50 Departement for Intensive Care Medicine, Kantonsspital Nidwalden, Stans, Switzerland; 51 Intensivstation, Spital Simmental-Thun-Saanenland AG, Thun, Switzerland; 52 Klinik für Anaesthesie und Intensivmedizin, Spitalzentrum Oberwallis, Visp, Switzerland; 53 Service d’Anesthesiologie, EHNV, Yverdon-les-Bains, Switzerland; 54 Interdisziplinaere Intensivstation, Stadtspital Triemli, Zurich, Switzerland; 55 Abteilung für Anaesthesiologie und Intensivmedizin, Hirslanden Klinik Im Park, Zurich, Switzerland; 56 Institut für Anaesthesiologie und Intensivmedizin, Klinik Hirslanden, Zurich, Switzerland; 57 General Surgery, Samsun Training and Research Hospital, Samsun, Turkey; 58 Soins intensifs, Groupement Hospitalier de l’Ouest Lémanique, Hôpital de Nyon, Nyon, Switzerland; 59 Department of Intensive Care Medicine, Erasmus Medical Center, Rotterdam, Netherlands; 60 Department of Anesthesiology and Critical Care Medicine, University Hospital of Nancy, Nancy, France; 61 Servicio de Medicina intensiva, Hospital Verge de la Cinta, Carrer de les Esplanetes 44, 43500 Tortosa, Tarragona Spain

**Keywords:** COVID-19, ARDS, Respiratory support, Noninvasive mechanical ventilation, High flow oxygen therapy, Invasive mechanical ventilation, Standard oxygen therapy, Patient self-inflicted lung injury

## Abstract

**Background:**

Uncertainty about the optimal respiratory support strategies in critically ill COVID-19 patients is widespread. While the risks and benefits of noninvasive techniques versus early invasive mechanical ventilation (IMV) are intensely debated, actual evidence is lacking. We sought to assess the risks and benefits of different respiratory support strategies, employed in intensive care units during the first months of the COVID-19 pandemic on intubation and intensive care unit (ICU) mortality rates.

**Methods:**

Subanalysis of a prospective, multinational registry of critically ill COVID-19 patients. Patients were subclassified into standard oxygen therapy ≥10 L/min (SOT), high-flow oxygen therapy (HFNC), noninvasive positive-pressure ventilation (NIV), and early IMV, according to the respiratory support strategy employed at the day of admission to ICU. Propensity score matching was performed to ensure comparability between groups.

**Results:**

Initially, 1421 patients were assessed for possible study inclusion. Of these, 351 patients (85 SOT, 87 HFNC, 87 NIV, and 92 IMV) remained eligible for full analysis after propensity score matching. 55% of patients initially receiving noninvasive respiratory support required IMV. The intubation rate was lower in patients initially ventilated with HFNC and NIV compared to those who received SOT (SOT: 64%, HFNC: 52%, NIV: 49%, *p* = 0.025). Compared to the other respiratory support strategies, NIV was associated with a higher overall ICU mortality (SOT: 18%, HFNC: 20%, NIV: 37%, IMV: 25%, *p* = 0.016).

**Conclusion:**

In this cohort of critically ill patients with COVID-19, a trial of HFNC appeared to be the most balanced initial respiratory support strategy, given the reduced intubation rate and comparable ICU mortality rate. Nonetheless, considering the uncertainty and stress associated with the COVID-19 pandemic, SOT and early IMV represented safe initial respiratory support strategies. The presented findings, in agreement with classic ARDS literature, suggest that NIV should be avoided whenever possible due to the elevated ICU mortality risk.

**Supplementary Information:**

The online version contains supplementary material available at 10.1186/s13054-021-03580-y.

## Background

Coronavirus disease 2019 (COVID-19) has generated a surge of critically ill patients who require invasive mechanical ventilation (IMV) overburdening intensive care units (ICU) worldwide.

Traditionally, the treatment of acute respiratory distress syndrome (ARDS) has focused mainly on IMV and its optimization [1]; nonetheless, in the last decade new approaches have been increasingly explored, primarily high-flow oxygen therapy by nasal cannula (HFNC) and noninvasive positive-pressure ventilation (NIV) [2, 3]. At the onset of the COVID-19 pandemic, most clinicians supported by the recommendations of international guidelines employed either standard oxygen therapy (SOT) or early IMV for the treatment of COVID-19-induced ARDS (CARDS) [4]. This choice was probably influenced by the numerous uncertainties regarding the new pathology, but also to avoid endangering hospital personnel by generating aerosols with HFNC and NIV. Nonetheless, in certain areas and centers, a lack of mechanical ventilators and adequately trained ICU staff forced clinicians to use noninvasive techniques to treat CARDS [5].

The high mortality rate associated with CARDS observed at the start to the pandemic has decreased over time [6, 7]. While many factors may explain this improvement, the decision to use invasive or noninvasive respiratory support remains one of the most controversial ones [8]. Expert opinions range widely. While some eminent authors urge for early intubation at the first signs of respiratory fatigue, to prevent patient self-inflicted lung injury (P-SILI) [9–12], others argue that all noninvasive options should be exhausted before proceeding to IMV [13–18]. Nevertheless, there is a surprising lack of evidence regarding the optimal respiratory support strategy.

The present study was designed in the context of the ubiquitous uncertainty surrounding respiratory support strategies in critically ill COVID-19 patients. This study consists of a subanalysis of the data collected prospectively in the RISC-19-ICU registry [19]. The main objective was to determine which respiratory support strategy employed during the first months of the COVID-19 pandemic was associated with a better overall prognosis. To reflect the early intubation trend followed during the first months of the pandemic, patients directly intubated on ICU admission but with matched severity characteristics to the noninvasively supported patients were also included in the analysis, constituting an independent respiratory support strategy.

## Methods

This was a retrospective subanalysis of data from the prospective RISC-19-ICU registry, which contains a standardized dataset of all critically ill COVID-19 patients admitted to the collaborating centers during the ongoing pandemic.

The RISC-19-ICU registry was deemed exempt from the need for additional ethics approval and patient informed consent by the ethics committee of the canton of Zurich (KEK 2020-00322, ClinicalTrials.gov Identifier: NCT04357275). The present study complies with the tenets of the Declaration of Helsinki, the Guidelines on Good Clinical Practice (GCP-Directive) issued by the European Medicines Agency, as well as Swiss law and Swiss regulatory authority requirements. All collaborating centers have complied with all local legal and ethical requirements. As of October 1, 2020, 63 collaborating centers in 10 countries, were actively contributing to the RISC-19-ICU registry. For further specifications on the RISC-19-ICU registry structure and data collection, see Additional file 1: e-Appendix 1.

### Inclusion and exclusion criteria

Patients were included in the present substudy if they required SOT (≥10 L/min [20]), HFNC, NIV, or IMV at the time point of admission to the ICU defined as day 0. Patients without a full ICU outcome data set, with SOT <10 L/min, or with a do-not-intubate order at day 0 were excluded. For the days ensuing ICU admission, the daily respiratory support therapy was defined as the main strategy used during the chart day.

### Initial ventilation support group definitions

For study purposes, patients were categorized into four groups according to their maximal respiratory support at ICU admission (day 0), as follows: **(1) SOT group:** patients receiving SOT with an oxygen flow of ≥10 L/min (FiO_2_ was approximated based on the delivered oxygen flow as described by Farias et al. [21]); **(2) HFNC group:** patients receiving HFNC, defined as a device delivering humidified and heated oxygen at a flow rate above 30 L/min; **(3) NIV group:** patients receiving NIV, irrespective of interface, mode and ventilator type employed; and **(4) IMV group:** intubated patients receiving IMV.

### Statistical analysis

Missing data handling is described in Additional file 1: e-Appendix 2. Comparisons of population characteristics were performed using the analysis of variance or Kruskal–Wallis test, as appropriate, and the Chi-squared test for categorical variables. Nearest neighbor matching with a propensity score caliper distance of 0.1 was employed to select IMV patients with ICU admission characteristics comparable to those of the patients in the SOT, HFNC and NIV groups. Patients having received IMV in another institutions ICU before admission to the RISC-19-ICU center were excluded from the matching process. To enable comparability between IMV and the noninvasive respiratory support strategies, Sequential Organ Failure Assessment (SOFA) and Simplified Acute Physiology II (SAPS II) scores were used without the mechanical ventilation and neurologic sub-scores for the matching process. An optimal quality match was defined as a standardized mean difference (SMD) ≤0.1 per matching variable between patients in the IMV group and the other groups (SOT, HFNC and NIV) [22].

Univariable Cox proportional hazard models coupled to the Kaplan–Meier estimator were employed to analyze the effects, represented by hazard ratios (HR), of the different respiratory support strategies on the incidence of intubation, ICU mortality and discharge from ICU. Multivariable adjusted HRs were calculated for every model independently by means of an iterative, step-wise, maximum likelihood optimizing algorithm, controlling for collinearity, interactions, and effect size variation in every iteration. The maximum number of covariates per model was chosen to ensure 1 to 10 events per covariate. Comparison of survival distributions among the various respiratory support strategies was approached by means of the log-rank test. Proportional hazard assumptions were assessed through inspection of Schoenfeld residuals.

Generalized linear regression model (GLM) analysis, considering all recorded baseline characteristics at ICU admission, was employed to determine the best predictive model for mortality in patients initially receiving HFNC and NIV and requiring delayed IMV. Multivariable GLM analysis was performed by means of an iterative, step-wise, maximum likelihood optimizing algorithm initially considering all variables with *p*<0.1 on the univariable analysis. First-order interaction terms between the predictor variables were tested for all models, and excluded if not improving the final model fit. For the final GLM model, a prognostic score and nomogram were generated, and receiver operating characteristics (ROC) analysis was employed alongside minimal Euclidean distance fitting to the (0, 1) point to determine the optimal cut-off value for the generated score. 95% confidence intervals (CI) and p values comparing the prognostic score to classic severity scores were generated by means of the bootstrap percentile method.

Statistical analysis was performed through a fully scripted data management pathway using the R environment for statistical computing version 3.6 .1. Due to the observational, prospective nature of this cohort study no power calculations were performed. A two-sided *p* <0.05 was considered statistically significant. Values are given as medians with interquartile ranges (IQR) or counts and percentages as appropriate.

## Results

### Baseline and matching

**Fig. 1:**
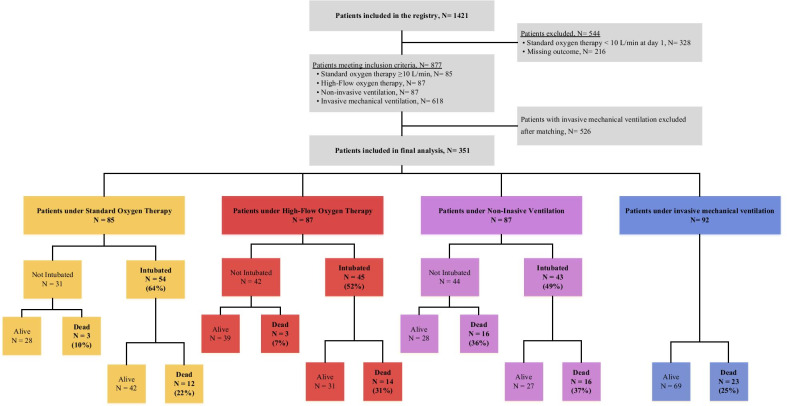
Study flowchart

Between March 13 and September 6, 2020, 1421 patients were included into the RISC-19-ICU registry. Of these 877 met the inclusion criteria at ICU admission (Fig. 1). During the first 24 hours of ICU stay, 618 (70%) patients had been intubated and were receiving mechanical ventilation; of the remaining 259 patients, 85 (10%) were being treated with SOT, 87 (10%) with HFNC and 87 (10%) with NIV. Compared to the other three groups, patients under IMV presented higher severity scores, including increased need for vasoactive medication (Additional file 1: e-Table 1).

**Table 1:** Demographics, characteristics at ICU admission, progression of respiratory support and outcome

	Overall *N* = 351	Standard oxygen therapy *N* = 85	High-flow oxygen therapy *N* = 87	Non-invasive positive-pressure ventilation *N* = 87	Invasive mechanical ventilation *N* = 92	*P* value
Centers that employed each strategy	49 (100)	28 (57)	21 (43)	26 (53)	35 (71)	
*Patient characteristics*						
Age, years	63 [55, 72]	63 [53, 72]	63 [55, 74]	66 [55, 76]	62 [55, 70]	0.454
Male gender	254 (73)	63 (75)	65 (75)	62 (71)	64 (71)	0.861
Body mass index, kg/m^2^	28 [25, 31]	28 [26, 32]	27 [25, 32]	26 [24, 29]	29 [26, 31]	0.029
Comorbidities	167 (47)	49 (57)	40 (46)	39 (44)	39 (42)	0.187
Ischemic heart disease	35 (10)	11 (12)	7 (8)	10 (11)	7 (7)	0.574
Diabetes mellitus	90 (25)	23 (27)	26 (29)	17 (19)	24 (26)	0.454
Chronic arterial hypertension	153 (43)	42 (49)	34 (39)	36 (41)	41 (44)	0.552
COPD	41 (11)	14 (16)	10 (11)	7 (8)	10 (10)	0.382
Immunosuppression†	40 (11)	7 (8)	13 (14)	7 (8)	13 (14)	0.311
*On ICU admission*						
Time from symptom onset to hospitalization, days	7 [4, 10]	7 [4, 10]	7 [3, 10]	6 [3, 10]	7 [4, 9]	0.797
Time from hospital admission to ICU, days	1 [0, 3]	1 [0, 3]	1 [0, 3]	2 [1, 4]	1 [0, 3]	0.233
APACHE score	11 [7, 18]	11 [7, 19]	10 [6, 13]	10 [7, 16]	11 [8, 20]	0.045
SAPS II score	38 [30, 59]	37 [30, 63]	35 [27, 44]	36 [29, 57]	45 [35, 63]	<0.001
SOFA score	7 [5, 8]	7 [4, 7]	6 [3, 7]	6 [4, 7]	7 [6, 8]	0.245
Vasopressors	32 (16)	9 (15)	5 (12)	11 (25)	7 (14)	0.341
Lactate, mmol/L	1.3 [0.9, 1.8]	1.1 [0.9, 1.5]	1.3 [0.9, 2]	1.5 [1, 1.8]	1.2 [0.9, 1.8]	0.352
FiO_2_, %	60 [50, 70]	60 [60, 60]	60 [44, 80]	60 [48, 70]	63 [45, 80]	0.180
Respiratory rate, 1/min	26 [22, 32]	28 [24, 32]	26 [22, 32]	28 [24, 37]	24 [21, 30]	0.052
SpO_2_, %	94 [91, 97]	92 [90, 94]	95 [92, 97]	94 [91, 97]	95 [92, 97]	0.118
PaO_2_/FiO_2_, mmHg	123 [92, 167]	117 [105, 160]	126 [79, 169]	135 [97, 168]	123 [90, 165]	0.612
CRP, mg/L	119 [33, 202]	153 [94, 217]	104 [31, 169]	111 [28, 202]	110 [23, 222]	0.052
Interleukin-6, ng/L	115 [56, 210]	153 [41, 236]	105 [82, 150]	111 [70, 175]	94 [50, 325]	0.968
D-dimer, µg/L	1146 [625, 2050]	1250 [653, 1899]	910 [505, 1628]	1394 [838, 5825]	1040 [638, 1905]	0.234
*Outcome*						
Requirement of intubation	234 (67)	54 (64)	45 (52)	43 (49)	92 (100)	<0.001
Withdrawal of life supporting therapies	51 (15)	14 (16)	8 (9)	15 (18)	14 (17)	0.408
ICU length of stay, days	13 [6, 23]	9 [3, 17]	13 [6, 24]	17 [8, 26]	15 [9, 24]	<0.001
ICU mortality	87 (25)	15 (18)	17 (20)	32 (37)	23 (25)	0.016

Values are given as median [interquartile range] or count (percent) as appropriate

*ICU* intensive care unit, *APACHE II* Acute Physiology And Chronic Health Evaluation II, *SAPS II* Simplified Acute Physiology Score II, *SOFA* Sequential Organ Failure Assessment, *FiO*_*2*_ Fraction Of Inspired O_2_, *SpO*_*2*_ Peripheral Oxygen Saturation, *PaO*_*2*_*/FiO*_*2*_* ratio* Partial Pressure of Arterial O_2_/Fraction Of Inspired O_2_, *CRP* C-reactive protein

^†^Immunosuppression was defined as any of the following: Hematologic malignancy, Human Immunodeficiency Virus, Hepatitis B or C infection, prescribed immunosuppressive medication

To allow for an unbiased assessment of respiratory strategies, a comparable population of IMV patients was extracted by propensity score matching against the other three groups based on 22 clinical, severity and laboratory parameters at admission (Additional file 1: e-Figure 1). After the matching process, 351 patients (85 SOT, 87 HFNC, 87 NIV and 92 IMV) were included in the final analysis. Matching quality was considered excellent, as reflected by an SMD ≤ 0.1 for all matching variables, excepting SAPS II (SMD = 0.13), bilirubin (SMD = 0.12), and mean arterial pressure (SMD = 0.11), in which the mean distributional difference between groups was nonetheless negligibly small (Additional file 1: e-Figure 1).

### Characteristics of the overall population

After the matching process, the baseline characteristics across all four groups at ICU admission were similar (Table 1, Additional file 1: e-Table 2). Patients were treated at 49 different ICUs, all of which followed different ventilation approaches. Until IMV was required or the patient could be weaned, no obvious crossovers between ventilation therapies seem to have been present (Additional file 1: e-Table 3). Further, there was no obvious temporal relationship between the period of the pandemic during which patients were admitted to the ICU and the use of a specific respiratory support strategy or mortality rate (Additional file 1: e-Figure 2 and e-Figure 3).

**Table 2:** Characteristics and disease progression in patients requiring invasive mechanical ventilation

	Overall *N* = 234	Standard oxygen therapy *N* = 54	High-flow oxygen therapy *N* = 45	Non-invasive positive-pressure ventilation *N* = 43	Invasive mechanical ventilation *N* = 92	*P* value
Time from hospital admission to intubation, days	2 [1, 5]	3 [1, 5]	3 [2, 6]	4 [3, 7]	1 [0, 3]	<0.001
Duration of noninvasive respiratory support, days	1 [1, 2]	1 [1, 1]	1 [1, 2]	1 [1, 2]	0 [0, 0]	0.109
SOFA score on intubation day	6 [5, 8]	6 [6, 8]	7 [6, 8]	6 [5, 7]	7 [6, 8]	0.258
*Ventilatory parameters after intubation*						
FiO_2_, %	60 [44, 75]	50 [40, 67]	60 [44, 67]	45 [41, 65]	63 [45, 80]	0.022
SpO_2_, %	94 [90, 97]	91 [88, 95]	94 [91, 96]	93 [89, 96]	96 [92, 97]	0.082
PaO_2_/FiO_2_, mmHg	137 [95, 179]	143 [94, 195]	136 [99, 181]	146 [108, 169]	123 [90, 165]	0.256
PaCO_2_, kPa	5.5 [4.7, 6.4]	5.8 [5.1, 6.7]	5.8 [4.7, 6.6]	5.7 [4.9, 6.4]	5.1 [4.3, 5.8]	0.002
Tidal volume/IBW, ml/kg	6.0 [5.6 , 7]	6.4 [5.7, 7.0]	6.0 [5.5, 6.7]	6.0 [5.6, 7.3]	6.1 [5.7, 7.1]	0.982
Respiratory rate, 1/min	24 [20, 28]	24 [20, 28]	21 [18, 27]	23 [20. 27]	24 [21, 30]	0.008
PEEP, cmH_2_O	12 [10, 12]	10 [10, 12]	10 [10, 12]	12 [10, 14]	12 [10, 14]	0.266
Plateau pressure, cmH_2_O	23 [21, 26]	24 [22, 25]	24 [22, 25]	24 [23, 27]	21 [21, 25]	0.838
Driving pressure, cmH_2_O	13 [10, 14]	13 [10, 14]	14 [12, 15]	13 [12, 14]	11 [10, 14]	0.96
Static compliance, ml/cmH_2_O	35 [26, 44]	34 [27, 44]	29 [25, 36]	28 [24, 34]	36 [31, 49]	0.603
*Treatment*						
Corticosteroids	50 (21)	11 (20)	13 (29)	12 (28)	14 (15)	0.194
*Organ failure and support during ICU stay*						
Prone positioning	148 (63)	35 (64)	31 (68)	27 (62)	55 (59)	0.764
Decrease in CRP from Day 0–7, %	27 [−77, 83]	19 [−100, 67]	17 [−27, 90]	21 [−103, 85]	42 [−48, 87]	0.02*
Vasopressors	187 (80)	45 (83)	38 (84)	39 (91)	65 (71)	0.029
Acute kidney injury	52 (22)	9 (17)	16 (36)	11 (26)	16 (17)	0.068
Renal replacement therapy	40 (17)	9 (17)	9 (20)	6 (14)	16 (17)	0.901
Tracheotomy	41 (17)	7 (13)	11 (24)	7 (16)	16 (17)	0.346
*Outcome*						
Withdrawal of life supporting therapies	38 (17)	11 (20)	6 (13)	7 (17)	14 (17)	0.857
ICU length of stay, days	16 [9, 26]	13 [7, 21]	21 [13, 29]	18 [9, 27]	15 [9, 24]	0.052
ICU mortality	65 (28)	12 (21)	14 (31)	16 (37)	23 (25)	0.342

Values are given as median [IQR] or count (percent) as appropriate

*ICU* intensive care unit, *SOFA* Sequential Organ Failure Assessment, *FiO*_*2*_ fraction of inspired O_2_, *SpO*_*2*_ peripheral oxygen saturation, *PaO*_*2*_*/FiO*_2_ ratio partial pressure of arterial O_2_/fraction of inspired O_2_, *PaCO*_*2*_ partial pressure of arterial CO_2_, *IBW* ideal body weight, *PEEP* positive end expiratory pressure, *CRP* C-reactive protein

^*^Calculated by means of mixed effect model analysis (Additional file 1: e-Table 2)

**Fig. 2:**
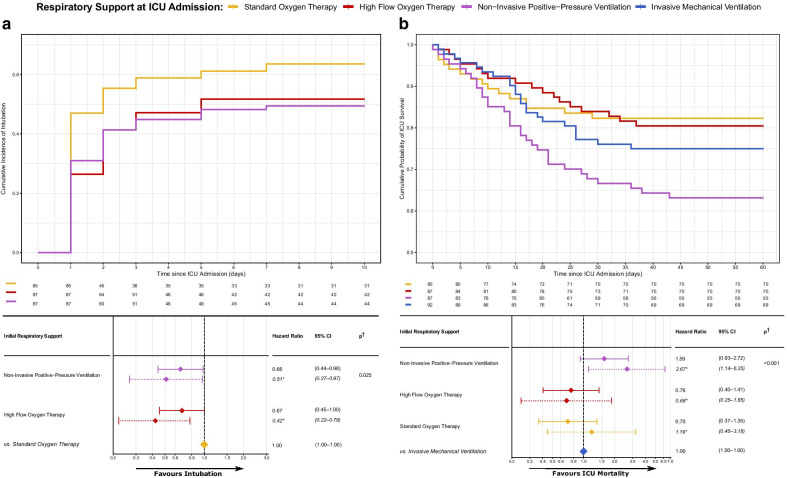
Kaplan–Meier curves for the **a** incidence of intubation and **b** intensive care unit mortality stratified by respiratory support strategy at intensive care unit admission. Forest plots reporting crude and multivariable adjusted (*italic) hazard ratios with 95% confidence intervals are displayed below the Kaplan–Meier curves for each respiratory support strategy. ^†^*p* values for between groups survival curve difference were calculated by means of the log-rank test

**Fig. 3:**
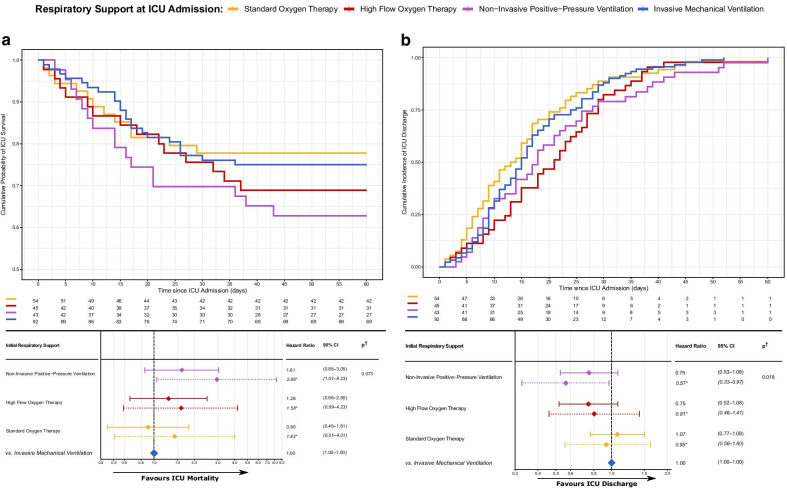
Kaplan–Meier curves for **a** intensive care unit mortality and **b** intensive care unit length of stay stratified by respiratory support strategy at intensive care unit admission (only intubated patients). Forest plots reporting crude and multivariable adjusted (*italic) hazard ratios with 95% confidence intervals are displayed below the Kaplan–Meier curves for each respiratory support strategy. ^†^*p* values for between groups survival curve difference were calculated by means of the log-rank test

Of the patients who were not intubated and invasively ventilated on ICU admission, 55% required intubation and IMV between the first and second day of their ICU stay. A smaller proportion of patients (*p* = 0.025) in the HFNC (52%) and NIV (49%) groups progressed toward delayed IMV, compared to those in the SOT group (64%) (Fig. 2a).

Overall, the ICU mortality rate was higher (*p* = 0.016) in patients initially ventilated with NIV than in the other groups (SOT: 18%, HFNC: 20%, NIV: 37%, IMV: 25%) (Fig. 2b). In patients who did not progress toward intubation, the ICU mortality rates were as follows: 10% in the SOT, 7% in the HFNC, and 36% in the NIV group (Additional file 1: e-Table 4). The amount of therapy withdrawals was similar between groups (*p* = 0.408).

### Characteristics of patients progressing toward intubation and invasive mechanical ventilation

The median duration of the in-hospital stay until intubation was longer (*p*<0.001) in the NIV group (4 [IQR, 3–7] days) compared to the other three groups (SOT: 3 [1–5] days, HFNC: 3 [2–6] days, IMV: 1 [0–3] days) (Table 2). At the day of intubation, patients progressing toward mechanical ventilation had an overall median partial pressure of arterial oxygen to inspired fraction of oxygen (P/F) ratio of 137 [95–179] mmHg, with no variations between groups (*p* = 0.256) (Table 2). In all groups the initial ventilator settings and static compliance were similar. The use of corticosteroids and prone positioning were also comparable between groups. Patients under early IMV experienced less pronounced C-reactive protein (CRP) dynamics, with a lower proportional peak increase and a larger proportional decrease over the initial 7 days of ICU stay compared to patients in the noninvasive respiratory support groups (*p* = 0.02) (Additional file 1: e-Figure 4; Additional file 1: e-Table 5). Patients who received initial NIV therapy had a greater need for vasopressors during the ICU stay (*p* = 0.029).

ICU mortality in patients requiring IMV was 28% (65) with a median length of stay of 16 [9–26] days. Patients initially treated with NIV who progressed toward IMV presented a trend (*p* = 0.073) toward higher ICU mortality (37%) as opposed to patients in the other groups (SOT: 21%, HFNC: 31%) when compared to the early IMV group (25%) (Fig. 3a). Patients who were initially treated with HFNC and NIV, and later required IMV, had longer (*p* = 0.018) ICU lengths of stay than patients under initial SOT when compared to early IMV (Fig. 3b).

After multivariable adjustment for covariates, NIV was independently associated with a higher overall ICU mortality (adjusted HR 2.67, 95% CI [1.14–6.25]) as well as with an increased ICU mortality rate (adjusted HR 2.96, 95% CI [1.07–8.23]) and a prolonged length of ICU stay (adjusted HR 0.57, 95% CI [0.33–0.97]) in patients failing NIV and requiring delayed IMV, as opposed to the other respiratory support strategies (Figs. 2b, 3a, b; Additional file 1: e-Figures 5–8).

### Predictors of mortality in patients initially treated with HFNC or NIV patients with delayed intubation and invasive mechanical ventilation

To identify the HFNC and NIV patients with the worst ICU outcomes after progression to intubation and IMV, an iterative, multivariable GLM analysis was performed. The model identified age, respiratory rate and diagnosis of diabetes mellitus as independent prognostic factors of mortality (Additional file 1: e-Table 6; Additional file 1: e-Figure 9A). A prognostic score, based on the previously described model, presented a moderate prognostic ability (area under the receiver operating curve: 0.75, 95% CI [0.63–0.85]) for ICU mortality in these patients. This prognostic score was superior to all other tested prognostic scores at ICU admission (Additional file 1: e-Figure 9B; Additional file 1: e-Table 9). The Kaplan–Meier estimator presented in Additional file 1: e-Figure 9C shows the excellent (*p*<0.0001) dichotomizing capacity of a prognostic score of 134 points (Positive Likelihood Ratio for Mortality: 2.4) to identify patients with a higher risk of ICU mortality.

## Discussion

In this subpopulation of a prospective, critically ill COVID-19 cohort during the first peak of the pandemic, 70% of patients were intubated and mechanically ventilated on the day of admission to the ICU. Use of SOT, HFNC and NIV was limited to 10% of the patients, respectively. The incidence of intubation and IMV in patients initially supported with HFNC and NIV was 12–15% lower than in patients with SOT. Compared to the other respiratory support strategies, NIV was associated with higher ICU mortality rates. A prognostic score considering age, respiratory rate and diabetes mellitus at ICU admission performed moderately in identifying HFNC and NIV patients with increased mortalities after delayed intubation and may help to discern patients who are at lower risk for increased ICU mortality during a HFNC or NIV trial.

International guidelines in place at the onset of the pandemic recommended early IMV for critically ill COVID-19 patients; HFNC and NIV were not recommended, mainly due to safety concerns related to the production of aerosols, which could jeopardize the health of hospital staff [23]. Notwithstanding those recommendations, the proportion of patients ventilated with noninvasive respiratory support strategies in this study was comparable to that described in the setting of the LUNGSAFE study, in which 15% of patients received noninvasive respiratory support [24]. Numerous COVID-19 cohort studies conducted in Europe and the United States have described similar proportions of noninvasive support measures [7, 18, 25–27].

Although contradictory results have been reported regarding the value of HFNC to avoid intubation [20, 28], this technique has been shown to reduce mortality rates in cases of acute hypoxemic failure [20], thus finding its place in international respiratory support recommendations [29]. In critically ill COVID-19 patients, other studies have shown—consistent with the data presented in our study—lower intubation and IMV rates, but without any reduction in ICU mortality [30]. The initially postulated risk of virus aerosolisation can probably be minimized by using conventional type I surgical masks over the nasal cannula [31]. By contrast, NIV remains controversial in the treatment of ARDS, a debate that is evident in the absence of unambiguous recommendations in clinical guidelines [32]. Although the use of NIV has been correlated with a reduced need for IMV and lower mortality rates in mild ARDS [33], the available evidence in severer expressions of ARDS indicates higher mortality rates [20, 24, 34]. In ARDS of viral etiology especially, the use of NIV is associated with high failure rates (up to 85%) [35].

Patients may—in an attempt to maintain homeostasis—initiate a vicious cycle through vigorous breathing efforts, exacerbating their lungs pathology by means of extremely elevated transpulmonary forces, leading to excessive stress and increased pulmonary inflammation [36, 37]. In our study, this patient-induced biotrauma might be one of the factors explaining the pronounced CRP dynamics in the noninvasively supported groups as opposed to those receiving early IMV [38]. Consequently, the prolonged use of noninvasive ventilation, delaying intubation in patients who ultimate fail and thus require IMV, has been associated with higher mortality rates in ARDS [39–43], as well as in critically ill COVID-19 patients [44–46]. The excess mortality observed in patients treated with NIV in this study might thus be explained by the longer period of harmful spontaneous breathing in patients failing NIV therapy, exacerbated by an increased respiratory rate and disproportionate tidal volumes induced by NIV therapy [24, 47].

If faced with a choice, physicians will intuitively prioritize avoidance of intubation and IMV, provided that this strategy does not imply any increase in mortality risk. Thus, the data presented in this study suggests that the best strategy appears to be an initial closely monitored HFNC trial with thorough assessment of clinical improvement, followed by proactive intubation and IMV in patients with a high risk of failure and mortality. The use of prognostic scores, such as the one exemplified in this study, may support clinical decision making to differentiate between patients who are treatable with noninvasive respiratory support strategies from those likely to have a worse outcome if intubation is delayed [48, 49]. To which degree static scores or dynamic scores taking advantage of the temporal assessment of patients, such as the ROX score, may improve ICU outcome nevertheless remains to be assessed [18, 49].

The present study has several limitations. First, the lack of randomization between respiratory support groups and it being a retrospective analysis, lead to many possible outcome modifying biases, such as the inability to assess the influence of human and material resources on treatment outcomes. Nonetheless, the lack of randomization was minimized through the application of propensity score matching to numerous variables at ICU admission, thus ensuring the comparability of the study groups in terms of the most objectively assessable patient characteristics. Second, the lack of a universal respiratory support protocol implies a high level of center- and clinician-related variability and prevents a mechanistic reasoning behind the described effects. On the other hand, the observational nature of this study potentially reflects the clinicians’ expertise more than a protocolized, randomized four-arm study, thereby reducing bias caused by variations in clinical experience or disfavour of a specific type of respiratory support strategy. Consequently the present study offers a representative view of the respiratory support strategies employed during the first peak of the pandemic. Third, some of the crude mortality trends observed in this study lacked statistical significance. However, given the moderate numbers of patients in each ventilation strategy, the large number of centers, the lack of a centralized protocol, and the statistical significance in the adjusted analyses, the observed signals provide a certain robustness for clinical decision-making and the development of hypotheses for future confirmatory, controlled studies. Fourth, the available registry data did not allow to determine the time on IMV for all patients, thus preventing an analysis of ventilator-free time. Fifth, the data underlying the prognostic score analysis were assessed on a daily basis, thus diminishing the prognostic capacity for scores, which require higher temporal resolution. Finally, the here proposed prognostic score has not been validated in other NIV and HFNC populations, thus caution is advised when employing it in a clinical framework; external validation is warranted.

## Conclusion

Given that patients who received HFNC in this cohort had lower intubation rates but comparable ICU mortality, the most reasonable initial ventilation strategy in critically ill COVID-19 patients appears to be a closely monitored trial of HFNC, prioritizing rapid intubation and IMV in patients with a high risk of failure. Nonetheless, considering the highly uncertain and stressful clinical setting experienced during the first wave of the COVID-19 pandemic, SOT and early IMV both represent safe and “cautious” initial respiratory support strategies. The presented findings, in agreement with classic ARDS literature, suggest that NIV should be avoided whenever possible due to an associated elevated ICU mortality risk.

## Supplementary Information

**Additional file 1.** **e-Appendix 1:** Specifications on the RISC-19-ICU registry structure and data collection. **e-Appendix 2:** Missing data handling. **e-Table 1:** Overall “unmatched” baseline characteristics on Day 0. **e-Figure 1:** “Love Plot” presenting Standardized Mean Differences between the unmatched and matchedcohort. **e-Table 2:** Demographics, characteristics at ICU admission, progression of respiratory support and outcome; stratified by survivor status. **e-Table 3:** Progression of respiratory support stratified by respiratory support strategy at ICU admission. **e-Figure 2:** Temporal relationship between the period of admission to the intensive care unit and the use of respiratory support strategies or mortality rate. **e-Figure 3:** Kaplan Meier curves for intensive care unit mortality stratified by the period of admission to the intensive care unit. **e-Table 4:** Demographics, characteristics at ICU admission, progression of respiratory support and outcome for patients never requiring intubation and invasive mechanical ventilation. **e-Figure 4:** C-Reactive Protein stratified by respiratory support strategy on Day 0 over the first week of ICU stay. **e-Table 5:** Mixed Effect Model of C-Reactive Protein stratified by respiratory support strategy on Day 0 over the first week of ICU stay. **e-Figure 5:** Multivariable adjusted COX regression model for the incidence of intubation. **e-Figure 6:** Multivariable adjusted COX regression model for overall ICU mortality. **e-Figure 7:** Multivariable adjusted COX regression model for ICU mortality (intubated patients only). **e-Figure 8:** Multivariable adjusted COX regression model for ICU discharge (intubated patients only). **e-Table 6:** Prognostic Model for the identification of patients with lower ICU mortality risk after a failed HFNC or NIV trial. **e-Figure 9:** Nomogram, Receiver Operating Curves and stratified Kaplan Meier curve for a prognostic model identifying patients with lower ICU mortality risk after a failed HFNC or NIV trial. **e-Table 7:** Area Under the Receiver Operating Curves (AUROCs) for the Prognostic Score versus classic severity scores.

## Data Availability

Any intensive care unit or other center treating critically ill COVID-19 patients is invited to join the RISC-19-ICU registry at https://www.risc-19-icu.net. While the registry protocol prevents the deposition of the full registry dataset in a third-party repository, analyses on the full dataset may be requested by any collaborating center after approval of the study protocol by the registry board. Reproducibility of the results in the present study was ensured by providing code for registry-specific data transformation and statistical analysis for collaborative development on the GitHub and Zenodo repositories. The registry protocol and data dictionary is publicly accessible at https://www.risc-19-icu.net.

## Abbreviations

ARDS: Acute respiratory distress syndrome
CI: Confidence interval
CARDS: COVID-19 ARDS
COVID-19: Coronavirus disease 2019
CRP: C-reactive protein
GLM: Generalized linear regression model
HR: Hazard ratio
HFNC: High-flow oxygen therapy by nasal cannula
ICU: Intensive care unit
IMV: Invasive mechanical ventilation
IQR: Interquartile range
NIV: Noninvasive positive-pressure ventilation
P/F: Partial pressure of arterial oxygen to inspired fraction of oxygen
P-SILI: Patient self-inflicted lung injury
ROC: Receiver operating characteristics
SOT: Standard oxygen therapy
SMD: Standardized mean difference
SOFA: Sequential Organ Failure Assessment
SAPS II: Simplified Acute Physiology Score II
STROBE: Strengthening the Reporting of Observational Studies in Epidemiology
