# Supplementary material for: Implications of early respiratory support strategies on disease progression in critical COVID-19: a matched subanalysis of the prospective RISC-19-ICU cohort
Source: Crit Care. 2021 May 25;25:175. doi: 10.1186/s13054-021-03580-y (PMC8146172; doi:10.1186/s13054-021-03580-y)
Supplement: Supplementary file 1 — Additional file 1. e-Appendix 1: Specifications on the RISC-19-ICU registry structure and data collection. e-Appendix 2: Missing data handling. e-Table 1: Overall “unmatched” baseline characteristics on Day 0. e-Figure 1: “Love Plot” presenting Standardized Mean Differences between the unmatched and matchedcohort. e-Table 2: Demographics, characteristics at ICU admission, progression of respiratory support and outcome; stratified by survivor status. e-Table 3: Progression of respiratory support stratified by respiratory support strategy at ICU admission. e-Figure 2: Temporal relationship between the period of admission to the intensive care unit and the use of respiratory support strategies or mortality rate. e-Figure 3: Kaplan Meier curves for intensive care unit mortality stratified by the period of admission to the intensive care unit. e-Table 4: Demographics, characteristics at ICU admission, progression of respiratory support and outcome for patients never requiring intubation and invasive mechanical ventilation. e-Figure 4: C-Reactive Protein stratified by respiratory support strategy on Day 0 over the first week of ICU stay. e-Table 5: Mixed Effect Model of C-Reactive Protein stratified by respiratory support strategy on Day 0 over the first week of ICU stay. e-Figure 5: Multivariable adjusted COX regression model for the incidence of intubation. e-Figure 6: Multivariable adjusted COX regression model for overall ICU mortality. e-Figure 7: Multivariable adjusted COX regression model for ICU mortality (intubated patients only). e-Figure 8: Multivariable adjusted COX regression model for ICU discharge (intubated patients only). e-Table 6: Prognostic Model for the identification of patients with lower ICU mortality risk after a failed HFNC or NIV trial. e-Figure 9: Nomogram, Receiver Operating Curves and stratified Kaplan Meier curve for a prognostic model identifying patients with lower ICU mortality risk after a failed HFNC or NIV trial. e-Table 7: Area Under [file 13054_2021_3580_MOESM1_ESM.docx]

**Online Supplementary Material**

**Implications of early respiratory support strategies on disease progression in critical COVID-19:
A matched subanalysis of the prospective RISC-19-ICU cohort**

*Wendel Garcia PD., Aguirre-Bermeo H., Buehler PK. et al.*

- **e-Appendix 1:** Specifications on the *RISC-19-ICU* registry structure and data collection.
- **e-Appendix 2:** Missing data handling
- **e-Table 1:** Overall “unmatched” baseline characteristics on Day 0.
- **e-Figure 1:** *“Love Plot”* presenting Standardized Mean Differences between the unmatched and matched cohort.
- **e-Table 2:** Demographics, characteristics at ICU admission, progression of respiratory support and outcome; stratified by survivor status
- **e-Table 3:** Progression of respiratory support stratified by respiratory support strategy at ICU admission.
- **e-Figure 2:** Temporal relationship between the period of admission to the intensive care unit and the use of respiratory support strategies or mortality rate.
- **e-Figure 3:** Kaplan Meier curves for intensive care unit mortality stratified by the period of admission to the intensive care unit.
- **e-Table 4:** Demographics, characteristics at ICU admission, progression of respiratory support and outcome for patients never requiring intubation and invasive mechanical ventilation
- **e-Figure 4:** C-Reactive Protein stratified by respiratory support strategy on Day 0 over the first week of ICU stay.
- **e-Table 5:** Mixed Effect Model of C-Reactive Protein stratified by respiratory support strategy on Day 0 over the first week of ICU stay.
- **e-Figure 5:** Multivariable adjusted COX regression model for the incidence of intubation.
- **e-Figure 6:** Multivariable adjusted COX regression model for overall ICU mortality.
- **e-Figure 7:** Multivariable adjusted COX regression model for ICU mortality (intubated patients only).
- **e-Figure 8:** Multivariable adjusted COX regression model for ICU discharge (intubated patients only).
- **e-Table 6:** Prognostic Model for the identification of patients with lower ICU mortality risk after a failed HFNC or NIV trial.
- **e-Figure 9:** Nomogram, Receiver Operating Curves and stratified Kaplan Meier curve for a prognostic model identifying patients with lower ICU mortality risk after a failed HFNC or NIV trial.
- **e-Table 7:** Area Under the Receiver Operating Curves (AUROCs) for the Prognostic Score versus classic severity scores

**e-Appendix 1:** Specifications on the *RISC-19-ICU* registry structure, data collection and clinical definitions.

## Registry structure

A standardized dataset was prospectively collected during the ongoing COVID-19 pandemic for all critically ill COVID-19 patients admitted to the collaborating centres. Inclusion criteria for the *RISC-19-ICU* registry were (I) a laboratory confirmed SARS-CoV-2 infection by nucleic acid amplification according to the WHO-issued testing guidelines, and (II) critical manifestation of COVID-19 requiring treatment in an ICU or intermediate care unit, defined as a hospital ward specialized in the care of critically ill patients with the availability of organ support therapies including invasive mechanical ventilation and/or non-invasive ventilation. The data was collected through an anonymized electronic case report form managed by the REDCap electronic data capture tool hosted on a secure server by the Swiss Society of Intensive Care Medicine.

## Data collection

Data were collected on the day of ICU admission defined as day zero, and on days one, two, three, five, seven, up to 25 days thereafter, as well as the final outcome. Days were defined as ICU chart days, starting at 6:00 a.m. and spanning until 5:59 a.m. on the next day. Data contained in the registry included patient characteristics, treatment modalities and organ support therapies, including the use of mechanical ventilation, prone positioning, vital parameters, arterial blood gas analyses, and laboratory values such as inflammatory, coagulation, renal, liver, cardiac, and other relevant parameters. To allow a prospective collection of data in the on-going pandemic, even in centres with reduced research resources, an abbreviated and an extensive version of the eCRF were designed.

**Outcome**

**Day 11**

**Day 0**

**Day 1**

**Day 2**

**Day 3**

**Day 5**

**Day 7**

**Day 9**

**Day 25**

**ICU Stay**

**ICU Admission**

**ICU Discharge**

**Every 2 days…**

Clinical definitions

ARDS was defined according to the Berlin definition as acute, diffuse bilateral lung infiltrates of non-cardiac origin, characterized by hypoxemia with a PaO2/FiO2 ratio (P/F ratio) ≤ 300 mmHg under positive pressure respiratory support (≥5cmH_2_O positive end-expiratory airway pressure or continuous positive airway pressure)^1^. Acute kidney injury was diagnosed in accordance with the KDIGO criteria as either a serum creatinine increase to more than 1.5 x the baseline value, an absolute creatinine increase of ≥ 26.5 μmol/l, or a urine output of less than 0.5 ml/kg/h for 6–12 h^2^. Acute cardiac injury was defined according to the Fourth Universal Definition of Myocardial Infarction, as an elevation in high sensitivity cardiac Troponin levels above the 99th percentile, coupled to the existence of a dynamic change in said levels^3^. Bacteraemia and fungaemia were defined as positive blood cultures for a bacterial or fungal pathogen.

**e-Appendix 2:** Missing data handling

To account for missing data, multiple imputation by fully conditional specification with predictive mean matching was performed under the missing at random assumption on the full dataset^4^. The missing at random assumption was postulated after assessment for quasi-missingness at random through consideration of survival curves, the log-rank test and COX proportional hazards models, evaluating the interactions between variables presenting the highest missing rates and a possible pattern on diverging mortality rates, which was not patent^5^. All independent baseline variables recorded in the data set were included; for each variable a linear regression model accounting for all non-collinear and non-intercepting variables was specified. Five parallel imputation models with 100 iterations each were run. Quality of imputation models was assessed by analysis of mean and standard deviation convergence plots and comparison of distribution plots for every imputation model and imputed variable. Finally, for every model and variable t-tests and standard mean differences (SMD) between imputed and original distribution were calculated, with SMDs below 0.1 being regarded as optimal and above 0.2 as suboptimal, but acceptable, imputation^6,7^. Imputed data was employed for full-case matching and multivariable analysis. No outcome variables were imputed.

| **Variable** | **Missing (%)** | **Variable** | **Missing (%)** | **Variable** | **Missing (%)** | **Variable** | **Missing (%)** |
| --- | --- | --- | --- | --- | --- | --- | --- |
| Interleukin-6 | 80 | Neutrophils | 27 | Creatinine | 12 | FiO_2_ | 5 |
| LDH | 46 | Lymphocytes | 23 | Mean Arterial Pressure | 12 | pH | 5 |
| D-Dimer | 41 | Thrombocytes | 18 | HCO_3_ | 10 | paO_2_ | 4 |
| Bilirubin | 39 | CRP | 17 | Leucocytes | 10 | paCO_2_ | 4 |
| PCT | 37 | Respiratory Rate | 16 | Lactate | 8 | Potassium | 2 |


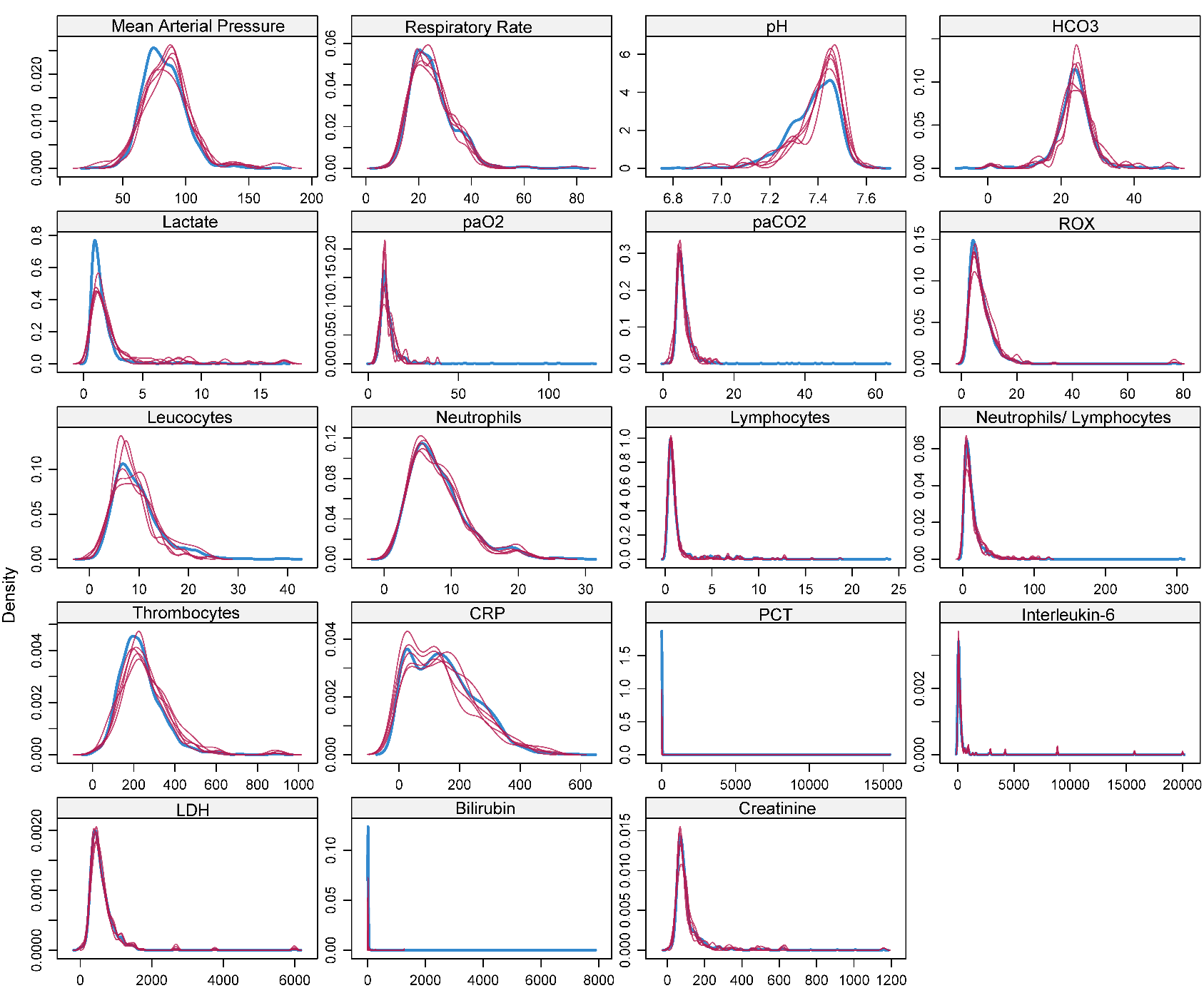


Distribution curves for imputed variables. Plotted in red are the distributions of the variables post imputation (5 models), as opposed to the original distribution which is plotted in blue.

**e-Table 1:** Overall “unmatched” baseline characteristics on Day 0. SOT - Standard Oxygen Therapy; HFNC - High Flow Nasal Cannula; NIV - Non-Invasive positive-pressure ventilation; IMV - Invasive Mechanical Ventilation.

|  | **Overall** | **SOT** | **HFNC** | **NIV** | **IMV** | **p** |
| --- | --- | --- | --- | --- | --- | --- |
| n | 877 | 85 | 87 | 87 | 618 |  |
| Age, years | 64 [56 - 72] | 63 [53 - 72] | 63 [55 - 74] | 66 [55 - 76] | 64 [57 - 72] | 0.809 |
| Male gender | 649 (74) | 63 (75) | 65 (76) | 62 (71) | 459 (75) | 0.909 |
| Body mass index, kg/m^2^ | 28 [25 - 31] | 28 [26 - 32] | 27 [25 - 32] | 26 [24 - 29] | 28 [25 - 31] | 0.032 |
| APACHE score | 17 [10 - 22] | 11 [7 - 19] | 10 [6 - 13] | 10 [7 - 16] | 19 [15 - 23] | <0.001 |
| SAPS II score | 60 [40 - 69] | 37 [30 - 63] | 35 [27 - 44] | 36 [29 - 57] | 65 [52 - 71] | <0.001 |
| SOFA score | 10 [6 - 12] | 7 [4 - 7] | 6 [3 - 7] | 6 [4 - 7] | 11 [9 - 13] | <0.001 |
| Vasopressors | 254 (50) | 9 (15) | 5 (12) | 11 (25) | 229 (64) | <0.001 |
| PaO_2_/FiO_2_, mmHg | 123 [87 - 171] | 117 [105 - 160] | 126 [79 - 169] | 135 [97 - 168] | 124 [85 - 173] | 0.622 |
| CRP, mg/L | 133 [52 - 218] | 153 [94 - 217] | 104 [31 - 169] | 111 [28 - 202] | 138 [60 - 221] | 0.013 |
| D-dimer, µg/L | 1456 [843 - 3440] | 1250 [653 - 1899] | 910 [505 - 1628] | 1394 [838 - 5825] | 1662 [899 - 3788] | 0.001 |
| Comorbidities | 458 (52) | 49 (58) | 40 (46) | 39 (45) | 330 (53) | 0.203 |
| Time from symptoms to hospital, days | 7 [4 - 10] | 7 [4 - 10] | 7 [3 - 10] | 6 [3 - 10] | 7 [4 - 9] | 0.833 |
| Time from hospital admission to ICU, days | 1 [0 - 3] | 1 [0 - 3] | 1 [0 - 3] | 2 [1 - 4] | 1 [0 - 4] | 0.190 |
| ICU length of stay, days | 14 [7 - 23] | 7 [3 - 17] | 13 [5 - 24] | 13 [6 - 23] | 15 [9 - 24] | <0.001 |
| ICU Mortality | 272 (31) | 15 (18) | 17 (20) | 32 (37) | 208 (34) | 0.001 |

**e-Figure 1:** *“Love Plot”* presenting Standardized Mean Differences between the unmatched and matched cohort.


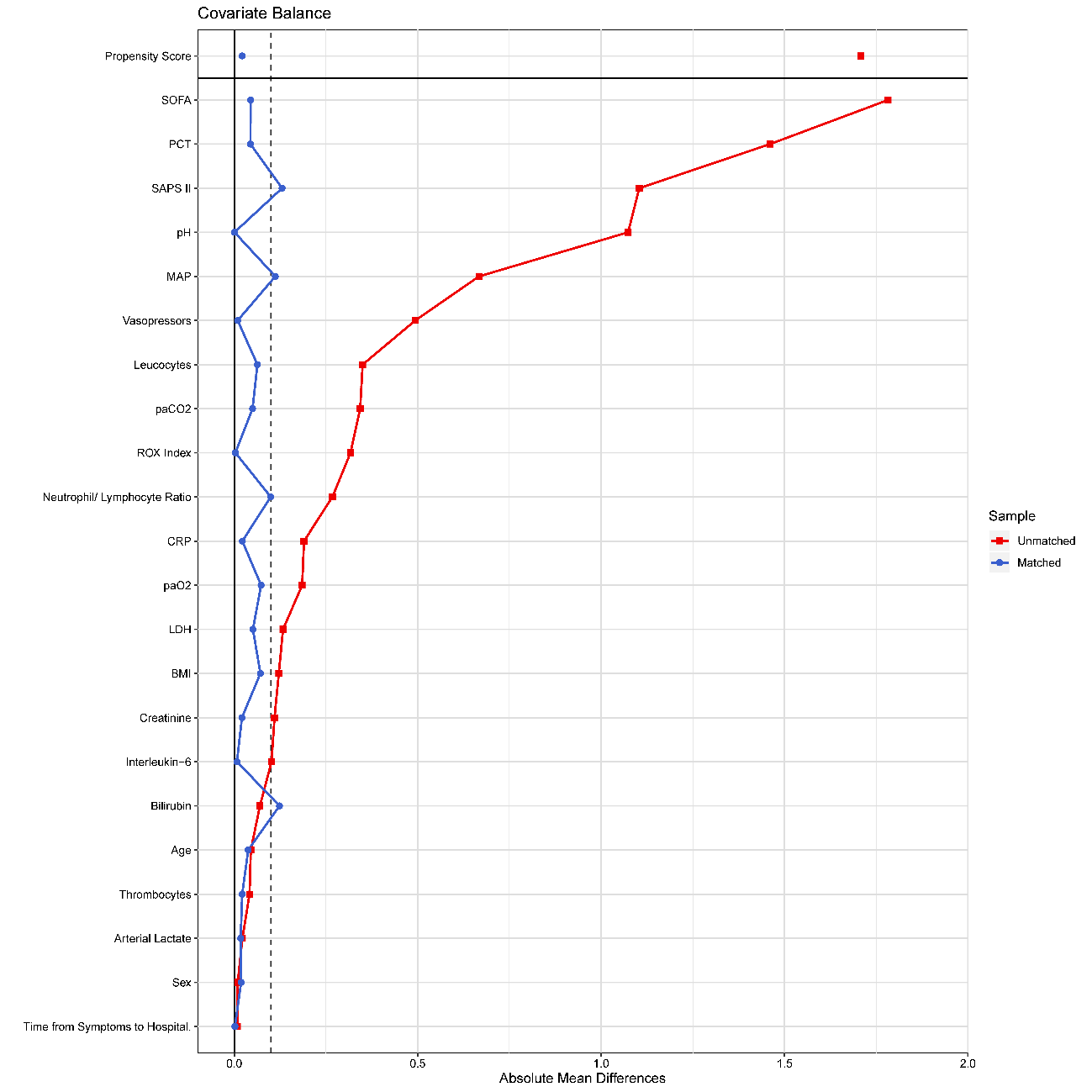


Difference in Standardized Mean Difference (SMD) between the unmatched groups (in red) and the matched groups (in blue). 22 variables were employed for the Propensity Score based Nearest Neighbor Matching with a 0.1 Caliper. An SMD value <0.1 is regarded as no difference in the variables values between two groups, and is an excellent parameter for Matching quality assessment.

Patients having received IMV in another institutions ICU before admission to the *RISC-19-ICU* center were excluded from the matching process. SOFA and SAPS II were employed without the mechanical ventilation and neurologic sub-score, to enable comparability between invasive mechanical ventilation and non-invasive respiratory support.

SOFA - Sequential Organ Failure Assessment; PCT - Procalcitonin; SAPS II - Simplified Acute Physiology Score II; MAP - Mean Arterial Pressure; paCO_2_ - partial pressure of arterial CO_2_; CRP - C-Reactive Protein; paO_2_ - partial pressure of arterial O_2_; LDH - lactate dehydrogenase; BMI - Body Mass Index; ROX Index – SpO2/ FiO2/ Respiratory Rate.

**e-Table 2:** Demographics, characteristics at ICU admission, progression of respiratory support and outcome; stratified by survivor status

|  | Overall  N= 351 | | Standard Oxygen Therapy  N= 85 | | High-Flow Oxygen Therapy  N= 87 | | Non- invasive positive-pressure ventilation  N= 87 | | Invasive mechanical ventilation  N= 92 | |
| --- | --- | --- | --- | --- | --- | --- | --- | --- | --- | --- |
|  | Survivors  N= 264 | Non-Survivors  N= 87 | Survivors  N= 70 | Non-Survivors  N= 15 | Survivors  N=70 | Non-Survivors  N= 17 | Survivors  N= 55 | Non-Survivors  N= 32 | Survivors  N= 69 | Non-Survivors  N= 23 |
| **Patients characteristics** |  |  |  |  |  |  |  |  |  |  |
| Age, years | 61 [53, 69] | 72 [63, 77] | 62 [52, 69] | 72 [67, 78] | 61 [53, 69] | 75 [73, 77] | 61 [52, 72] | 72 [64, 77] | 60 [56, 69] | 65 [55, 72] |
| Male gender | 185 (71) | 69 (79.3) | 51 (74) | 12 (80.0) | 54 (78) | 11 (64.7) | 33 (60) | 29 (90.6) | 47 (70) | 17 (73.9) |
| Body mass index, kg/m^2^ | 28 [25, 32] | 28 [25, 31] | 29 [27, 32] | 27 [24, 28] | 27 [25, 32] | 27 [25, 36] | 26 [24, 29] | 28 [25, 30] | 29 [25, 31] | 30 [26, 31] |
| Comorbidities | 114 (43) | 53 (61) | 40 (57) | 9 (60) | 30 (43) | 10 (59) | 17 (31) | 22 (69) | 27 (39) | 12 (52) |
| Ischemic heart  disease | 22 (8) | 13 (15) | 9 (13) | 2 (13) | 4 (6) | 3 (18) | 3 (6) | 7 (22) | 6 (9) | 1 (4) |
| Diabetes Mellitus | 56 (21) | 34 (39) | 17 (24) | 6 (40) | 18 (26) | 8 (47) | 5 (9) | 12 (38) | 16 (23) | 8 (35) |
| Chronic arterial  hypertension | 112 (42) | 41 (47) | 35 (50) | 7 (47) | 27 (39) | 7 (41) | 21 (38) | 15 (47) | 29 (42) | 12 (52) |
| COPD | 32 (12) | 9 (10) | 12 (17) | 2 (13) | 8 (11) | 2 (12) | 4 (7) | 3 (9) | 8 (12) | 2 (9) |
| Immuno-  suppression† | 27 (10) | 13 (15) | 6 (9) | 1 (7) | 8 (11) | 5 (29) | 4 (7) | 3 (9) | 9 (13) | 4 (17) |
| **On ICU admission** |  |  |  |  |  |  |  |  |  |  |
| Time from symptom onset to hospitalization, days | 7 [4, 10] | 7 [4, 10] | 7 [5, 10] | 5 [4, 8] | 7 [4, 10] | 6 [3, 9] | 6 [3, 11] | 6 [3, 10] | 6 [4, 8] | 8 [6, 13] |
| Time from hospital admission to ICU, days | 1 [0, 3] | 1 [0, 3] | 1 [0, 3] | 0 [0, 3] | 1 [0, 3] | 1 [0, 2] | 2 [0, 3] | 3.00 [1, 4] | 1 [0, 3] | 1 [0, 3] |
| APACHE score | 10 [6, 17] | 12 [10, 20] | 10 [7, 19] | 13 [10, 20] | 9 [5, 12] | 12 [10, 15] | 9 [6, 17] | 11 [8, 15] | 11 [7, 19] | 17 [10, 22] |
| SAPS II score | 36 [29, 58] | 43 [36, 61] | 35 [29, 63] | 48 [45, 66] | 32 [26, 40] | 43 [40, 46] | 34 [28, 59] | 37 [33, 45] | 43 [35, 62] | 53 [39, 67] |
| SOFA score | 7 [6, 10] | 7 [6, 9] | 8 [6, 9] | 8 [6, 9] | 6 [6, 9] | 6 [6, 8] | 7 [6, 10] | 6 [6, 10] | 8. [6, 11] | 7 [6, 10] |
| Lactate, mmol/L | 1.2 [0.9, 1.7] | 1.6 [1.2, 2.2] | 1.1 [0.9, 1.4] | 1.6 [1.3, 2.8] | 1.3 [0.9, 2.0] | 1.4 [1.0, 2.3] | 1.1 [0.9, 1.7] | 1.7 [1.4, 2.0] | 1.2 [0.9, 1.7] | 1.5 [1.2, 2.2] |
| FiO_2_, % | 60 [50, 70] | 60 [60, 80] | 60 [60, 60] | 60 [60, 60] | 60 [44, 88] | 70 [60, 80] | 50 [40, 70] | 60 [50, 74] | 60 [44, 80] | 70 [50, 83] |
| Respiratory rate, 1/min | 26 [22, 32] | 27 [22, 35] | 28 [25, 32] | 25 [24, 30] | 26 [22, 31] | 28 [21, 35] | 28 [24, 37] | 28 [24, 38] | 24 [21, 30] | 24 [22, 34] |
| SpO_2_, % | 92 [90, 98] | 93 [89, 98] | 92 [90, 97] | 92 [86, 97] | 92 [89, 97] | 93 [90, 95] | 92 [88, 98] | 92 [89, 99] | 93 [90, 98] | 93 [90, 98] |
| PaO_2_/FiO_2_, mmHg | 126 [95, 167] | 114 [87, 165] | 120 [107, 170] | 98 [87, 129] | 127 [79, 164] | 117 [78, 215] | 139 [104, 169] | 114 [97, 168] | 126 [89, 166] | 120 [93, 137] |
| CRP, mg/L | 122 [34, 210] | 111 [28, 178] | 153 [79, 241] | 145 [99, 178] | 104 [31, 177] | 64 [45, 153] | 96 [20, 196] | 112 [62, 220] | 115 [29, 224] | 34 [13, 148] |
| Procalcitonin, μg/l | 0.3 [0.2, 0.5] | 0.4 [0.2, 1.3] | 0.3 [0.2, 1.0] | 0.3 [0.1, 0.4] | 0.2 [0.1, 0.4] | 0.1 [0.1, 1.0] | 0.3 [0.2, 0.7] | 0.4 [0.3, 1.4] | 0.3 [0.1, 0.6] | 0.6 [0.2, 1.9] |
| Interleukin-6, ng/L | 129 [63, 207] | 91 [51, 211] | 149 [8, 233] | 185 [120, 332] | 126 [82, 170] | 82 [82, 82] | 124 [86, 179] | 100 [70, 156] | 98 [77, 637] | 50 [50, 240] |
| D-dimer, µg/L | 1242 [727, 2500] | 1520 [820, 3633] | 1250 [653, 2590] | 1250 [653, 1899] | 900 [675, 1854] | 910 [505, 1628] | 2267 [1327, 3775] | 1394 [838, 5825] | 1791 [1016, 2450] | 1791 [1058, 3743] |
| Bilirubin, μmol/l | 8 [4, 13] | 8 [1, 12] | 8 [6, 13] | 9 [8, 12] | 9 [5, 15] | 1 [1, 8] | 3 [1, 11] | 7 [1, 15] | 9 [7, 12] | 2 [0, 8] |
| Creatinine, μmol/l | 77 [63, 99] | 89 [68, 138] | 76 [63, 99] | 77 [70, 98] | 77 [67, 97] | 82 [67, 109] | 81 [63, 105] | 105 [71, 149] | 77 [61, 90] | 95 [65, 159] |
| **Outcome** |  |  |  |  |  |  |  |  |  |  |
| Requirement of intubation | 100 (38) | 42 (48) | 42 (60) | 12 (80) | 31 (44) | 14 (82) | 27 (49) | 16 (50) | 69 (100) | 23 (100) |
| Withdrawal of Life Supporting Therapies | 18 (7) | 33 (42) | 4 (6) | 10 (67) | 2 (3) | 6 (38) | 5 (9) | 10 (35) | 7 (11) | 7 (39) |
| ICU length of stay, days | 13 [6, 24] | 14 [8, 22] | 9 [3, 17] | 9 [3, 16] | 13 [6, 24] | 18 [8, 25] | 18 [8, 28] | 14 [8, 21] | 15 [9, 25] | 16 [10, 22] |
| Values are given as median [interquartile range] or count (percent) as appropriate.   ICU - Intensive Care Unit; APACHE II - Acute Physiology And Chronic Health Evaluation II; SAPS II - Simplified Acute Physiology Score II; SOFA - Sequential Organ Failure Assessment; FiO_2_ - Fraction Of Inspired O_2_; SpO_2_ - Peripheral Oxygen Saturation; PaO_2_/ FiO_2_ ratio - Partial Pressure Of Arterial O_2_/ Fraction Of Inspired O_2_; CRP - C-Reactive Protein.   †Immunosuppression was defined as any of the following: Hematologic malignancy, Human Immunodeficiency Virus, Hepatitis B or C infection, prescribed immunosuppressive medication. | | | | | | | | | | |

**e-Table 3:** Progression of respiratory support stratified by respiratory support strategy at ICU admission.

|  | Overall  N= 351 | Standard Oxygen Therapy  N= 85 | High-Flow Oxygen Therapy  N= 87 | Non- invasive positive-pressure ventilation  N= 87 | Invasive mechanical ventilation  N= 92 | P value |
| --- | --- | --- | --- | --- | --- | --- |
| **Progression of respiratory support** | | | | | | |
| SOT | 29 (8) | 29 (34) | 0 (0) | 0 (0) | 0 (0) |  |
| HFNC | 42 (12) | 1 (1) | 41 (47) | 0 (0) | 0 (0) |  |
| NIV | 46 (13) | 1 (1) | 1 (1) | 44 (51) | 0 (0) |  |
| IMV | 234 (67) | 54 (64) | 45 (52) | 43 (49) | 92 (100) | <0.001 |
| Values are given as count (percent). SOT - Standard Oxygen Therapy; HFNC - High Flow Nasal Cannula; NIV - Non-Invasive Ventilation; IMV - Invasive Mechanical Ventilation. | | | | | | |

**e-Figure 2:** Temporal relationship between the period of admission to the intensive care unit and the use of respiratory support strategies or mortality rate. SOT - Standard Oxygen Therapy; HFNC - High Flow Nasal Cannula; NIV - Non-Invasive positive-pressure ventilation; IMV - Invasive Mechanical Ventilation.

**
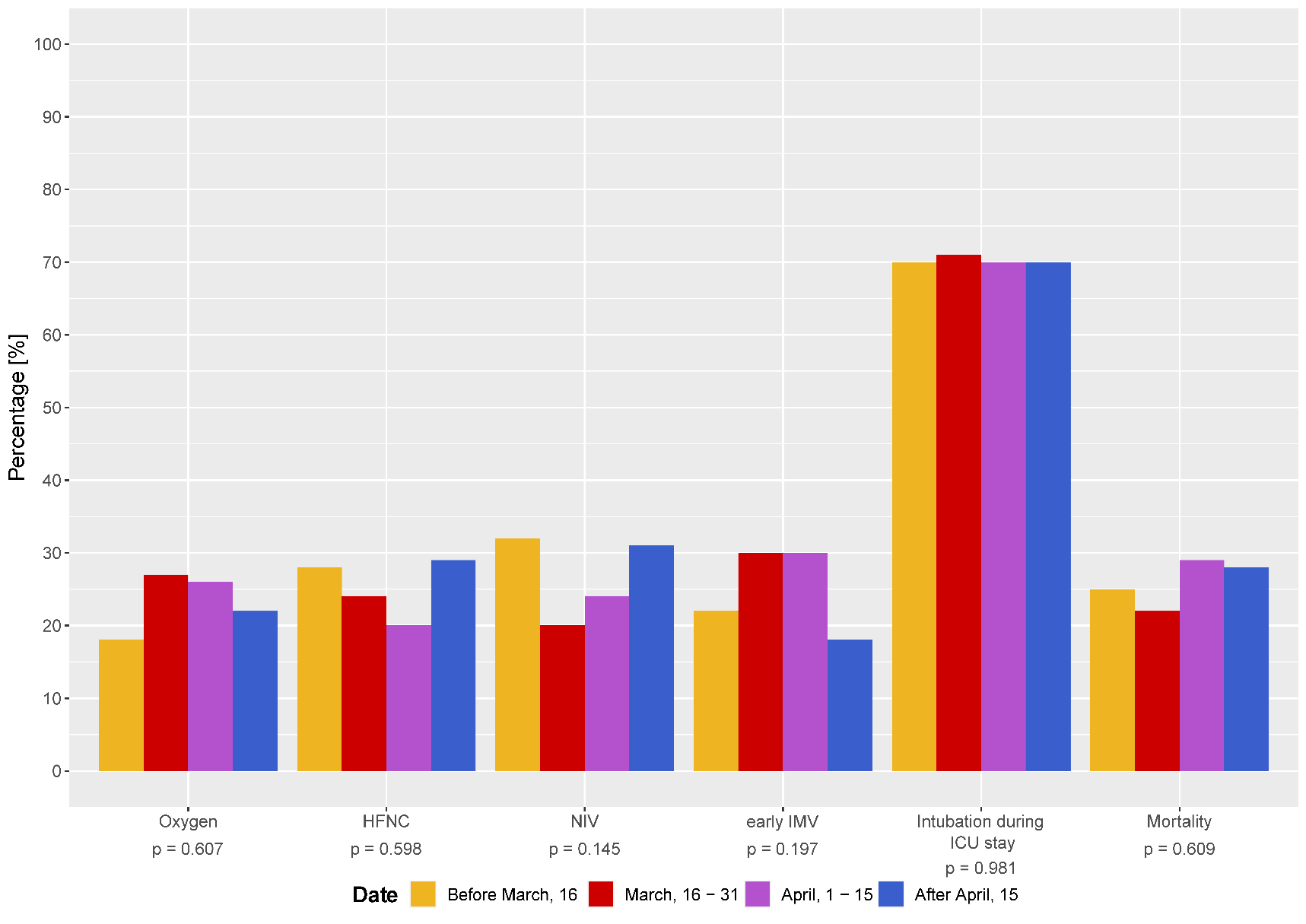
**

**e-Figure 3:** Kaplan Meier curves for intensive care unit mortality stratified by the period of admission to the intensive care unit.

**
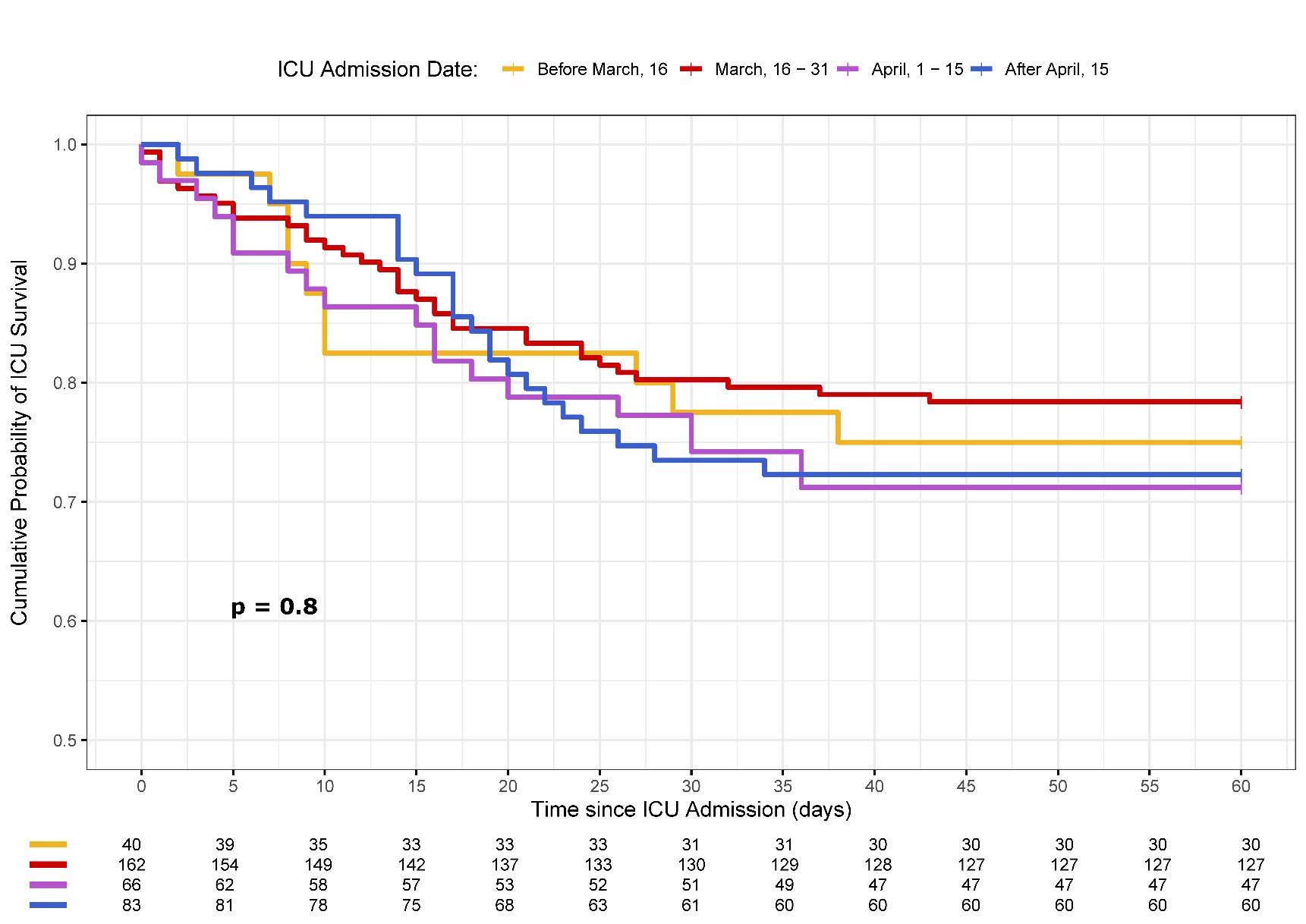
**

**e-Table 4:** Demographics, characteristics at ICU admission, progression of respiratory support and outcome for patients never requiring intubation and invasive mechanical ventilation

|  | Overall  N= 117 | Standard Oxygen Therapy  N= 31 | High-Flow Oxygen Therapy  N= 42 | Non- invasive positive-pressure ventilation  N= 44 | P value |
| --- | --- | --- | --- | --- | --- |
| **Patients characteristics** |  |  |  |  |  |
| Age, years | 63 [54, 75] | 66 [53, 77] | 60 [54, 69] | 66 [55, 77] | 0.401 |
| Male gender | 90 (78) | 24 (77) | 34 (83) | 32 (73) | 0.491 |
| Body mass index, kg/m^2^ | 27 [24, 30] | 29 [27, 31] | 26 [24, 29] | 25 [23, 29] | 0.008 |
| Comorbidities | 57 (49) | 20 (65) | 19 (45) | 18 (41) | 0.112 |
| Ischemic heart   disease | 15 (13) | 6 (19) | 3 (7) | 6 (14) | 0.298 |
| Diabetes Mellitus | 26 (22) | 11 (35) | 10 (24) | 5 (11) | 0.045 |
| Chronic arterial  hypertension | 47 (40) | 17 (55) | 15 (36) | 15 (34) | 0.150 |
| COPD | 14 (12) | 6 (19) | 5 (12) | 3 (7) | 0.257 |
| Immuno-  suppression^†^ | 13 (11) | 2 (6) | 7 (17) | 4 (9) | 0.337 |
| **On ICU admission** |  |  |  |  |  |
| Time from symptom onset to hospitalization, days | 7 [3, 10] | 7 [4, 10] | 7 [4, 10] | 6 [3, 12] | 0.924 |
| Time from hospital admission to ICU, days | 1 [0, 2] | 0 [0, 2] | 2 [0, 3] | 1 [0, 2] | 0.283 |
| APACHE score | 8 [6, 11] | 8 [6, 10] | 8 [5, 11] | 8 [5, 11] | 0.775 |
| SAPS II score | 30 [24, 36] | 32 [29, 36] | 29 [24, 36] | 30 [26, 37] | 0.540 |
| SOFA score | 6 [6, 9] | 6 [6, 8] | 6 [6, 10] | 6 [6, 9] | 0.548 |
| Lactate, mmol/L | 1.3 [0.9, 1.8] | 1.1 [1.0, 1.8] | 1.4 [1.1, 2.0] | 1.3 [0.9, 1.7] | 0.441 |
| FiO_2_, % | 60 [45, 60] | 60 [60, 60] | 50 [40, 68] | 50 [40, 60] | 0.020 |
| Respiratory rate, 1/min | 26 [22, 32] | 28 [23, 30] | 24 [22, 29] | 28 [24, 38] | 0.092 |
| SpO_2_, % | 92 [89, 98] | 92 [91, 98] | 92 [88, 96] | 92 [88, 97] | 0.537 |
| PaO_2_/FiO_2_, mmHg | 139 [106, 182] | 127 [106, 227] | 139 [106, 173] | 146 [106, 188] | 0.948 |
| CRP, mg/L | 108 [30, 198] | 140 [47, 204] | 91 [23, 168] | 122 [54, 212] | 0.428 |
| Procalcitonin, μg/l | 0.20 [0.10, 0.44] | 0.24 [0.15, 0.73] | 0.18 [0.07, 0.26] | 0.26 [0.18, 0.41] | 0.223 |
| Interleukin-6, ng/L | 70 [36, 154] | 52 [8, 120] | 150 [59, 232] | 70 [44, 128] | 0.372 |
| D-dimer, µg/L | 1010 [645, 2960] | 1155 [823, 2683] | 850 [632, 1666] | 1400 [600, 7500] | 0.503 |
| Bilirubin, μmol/l | 9 [4, 17] | 8 [6, 11] | 14 [8, 20] | 6 [1, 17] | 0.159 |
| Creatinine, μmol/l | 77 [67, 105] | 75 [64, 100] | 77 [67, 84] | 88 [70, 123] | 0.286 |
| **Outcome** |  |  |  |  |  |
| Withdrawal of Life Supporting Therapies | 13 (12) | 3 (10) | 2 (5) | 8 (19) | 0.155 |
| ICU length of stay, days | 6 [2, 13] | 3 [2, 6] | 7 [4, 12] | 11 [3, 24] | 0.001 |
| ICU mortality | 22 (19) | 3 (10) | 3 (7) | 16 (36) | 0.001 |
| Values are given as median [interquartile range] or count (percent) as appropriate.   ICU - Intensive Care Unit; APACHE II - Acute Physiology And Chronic Health Evaluation II; SAPS II - Simplified Acute Physiology Score II; SOFA - Sequential Organ Failure Assessment; FiO_2_ - Fraction Of Inspired O_2_; SpO_2_ - Peripheral Oxygen Saturation; PaO_2_/ FiO_2_ ratio - Partial Pressure Of Arterial O_2_/ Fraction Of Inspired O_2_; CRP - C-Reactive Protein.   †Immunosuppression was defined as any of the following: Hematologic malignancy, Human Immunodeficiency Virus, Hepatitis B or C infection, prescribed immunosuppressive medication. | | | | | |

**e-Figure 4:** C-Reactive Protein stratified by respiratory support strategy on Day 0 over the first week of ICU stay. Lines represent median values, shaded areas the interquartile ranges. SOT - Standard Oxygen Therapy; HFNC - High Flow Nasal Cannula; NIV - Non-Invasive positive-pressure ventilation; IMV - Invasive Mechanical Ventilation.


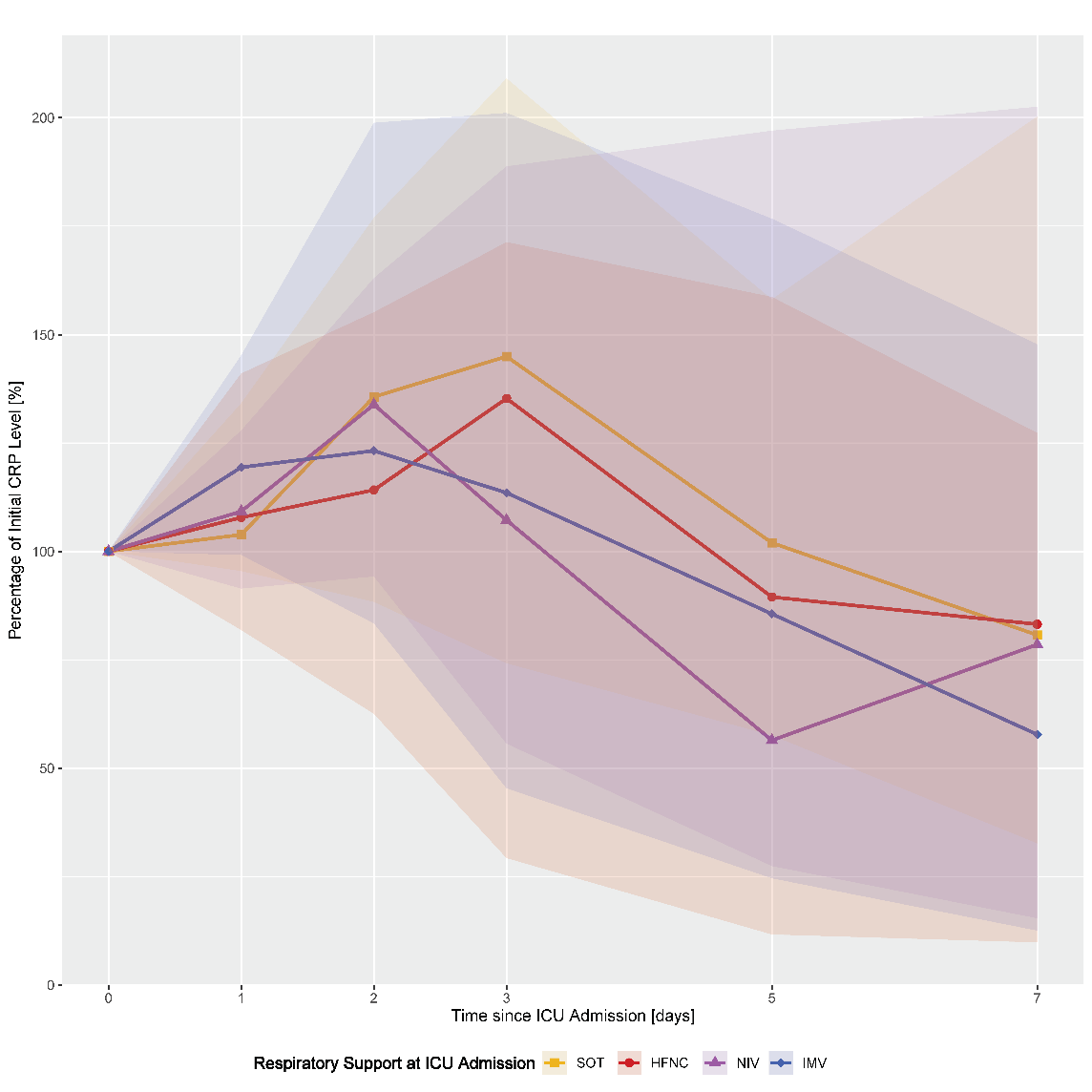


**e-Table 4:** Mixed Effect Model of C-Reactive Protein stratified by respiratory support strategy on Day 0 over the first week of ICU stay. SOT - Standard Oxygen Therapy; HFNC - High Flow Nasal Cannula; NIV - Non-Invasive positive-pressure ventilation; IMV - Invasive Mechanical Ventilation; CRP – C-reactive protein.

|  | **Day 0 - SOT** | **Day 0 - HFNC** | **Day 0 - NIV** | **Day 0 - IMV** | **Day 1 - SOT** | **Day 1 - HFNC** | **Day 1 - NIV** | **Day 1 - IMV** | **Day 2 - SOT** | **Day 2 - HFNC** | **Day 2 - NIV** | **Day 2 - IMV** |
| --- | --- | --- | --- | --- | --- | --- | --- | --- | --- | --- | --- | --- |
| **CRP, mg/l** | 157 [102 - 218] | 123 [58 - 160] | 88 [24 - 184] | 110 [23 - 222] | 171 [133 - 235] | 139 [40 - 195] | 113 [29 - 213] | 133 [70 - 261] | 211 [148 - 303] | 135 [41 - 226] | 177 [32 - 293] | 145 [59 - 265] |
| **Percentage of CRP at Day 0, %** | 100 [100 - 100] | 100 [100 - 100] | 100 [100 - 100] | 100 [100 - 100] | 104 [95 - 134] | 108 [82 - 141] | 109 [91 - 128] | 119 [99 - 145] | 136 [88 - 177] | 114 [63 - 155] | 134 [94 - 163] | 123 [83 - 199] |

|  | **Day 3 - SOT** | **Day 3 - HFNC** | **Day 3 - NIV** | **Day 3 - IMV** | **Day 5 - SOT** | **Day 5 - HFNC** | **Day 5 - NIV** | **Day 5 - IMV** | **Day 7 - SOT** | **Day 7 - HFNC** | **Day 7 - NIV** | **Day 7 - IMV** | **p - Group** | **p - Time** |
| --- | --- | --- | --- | --- | --- | --- | --- | --- | --- | --- | --- | --- | --- | --- |
| **CRP, mg/l** | 236 [99 - 302] | 190 [35 - 263] | 167 [11 - 298] | 103 [33 - 247] | 193 [78 - 267] | 98 [12 - 238] | 104 [7 - 227] | 69 [21 - 221] | 133 [42 - 306] | 106 [8 - 198] | 105 [6 - 211] | 59 [18 - 180] | 0.02 | < 0.0001 |
| **Percentage of CRP at Day 0, %** | 145 [74 - 209] | 135 [29 - 171] | 107 [56 - 189] | 114 [45 - 201] | 102 [58 - 158] | 89 [12 - 159] | 56 [27 - 197] | 86 [25 - 177] | 81 [33 - 200] | 83 [10 - 127] | 79 [15 - 203] | 58 [13 - 148] |  |  |

Statistical Analysis – Mixed Effect Model Analysis

For longitudinal analysis of C-reactive protein, differences between time points and initial respiratory support strategy were tested using linear mixed effects model analysis. As independent variable fixed effects, time point and initial respiratory support strategy were entered into the model, respectively, with and without interaction terms, which were retained only if they were found to contribute to the model. As random effects, intercepts for subjects as well as per-subject random slopes for the effect on dependent variables were employed. P values were calculated using a likelihood ratio test of the full model, with the effect in question, against a “null model”, without the effect in question.

**e-Figure 5:** Multivariable adjusted COX regression model for the incidence of intubation.


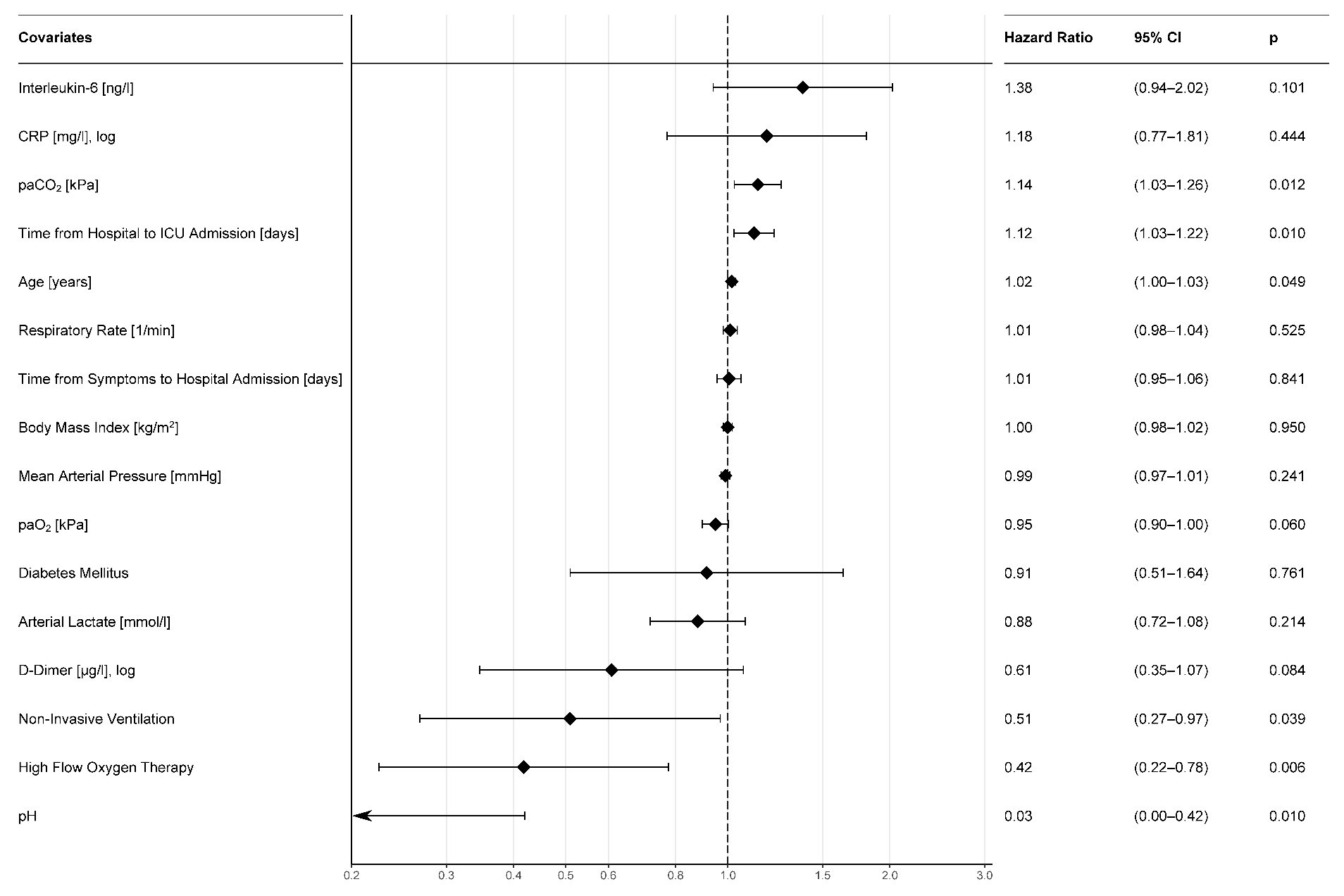


**e-Figure 6:** Multivariable adjusted COX regression model for overall ICU mortality.

**
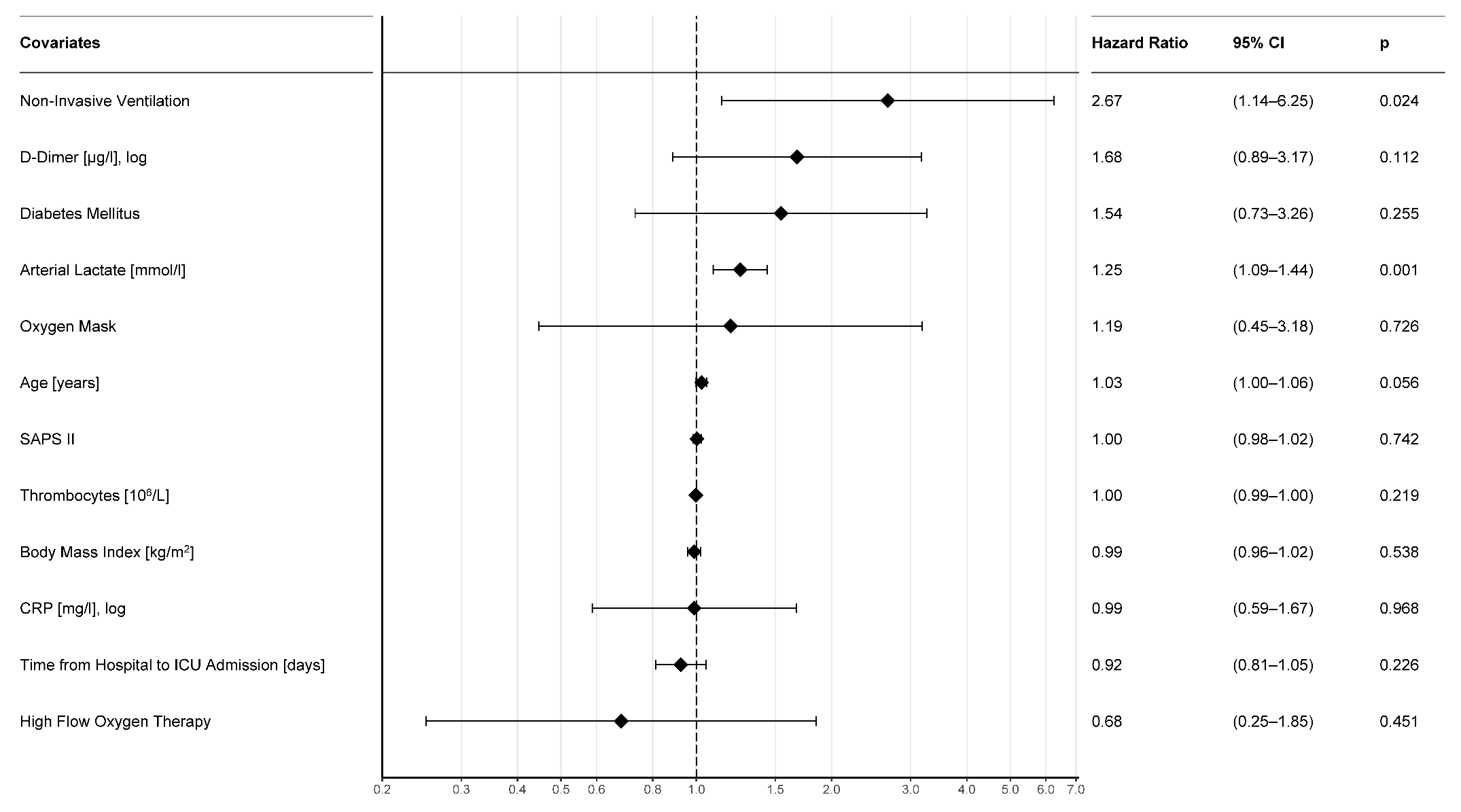
**

**e-Figure 7:** Multivariable adjusted COX regression model for ICU mortality (intubated patients only).


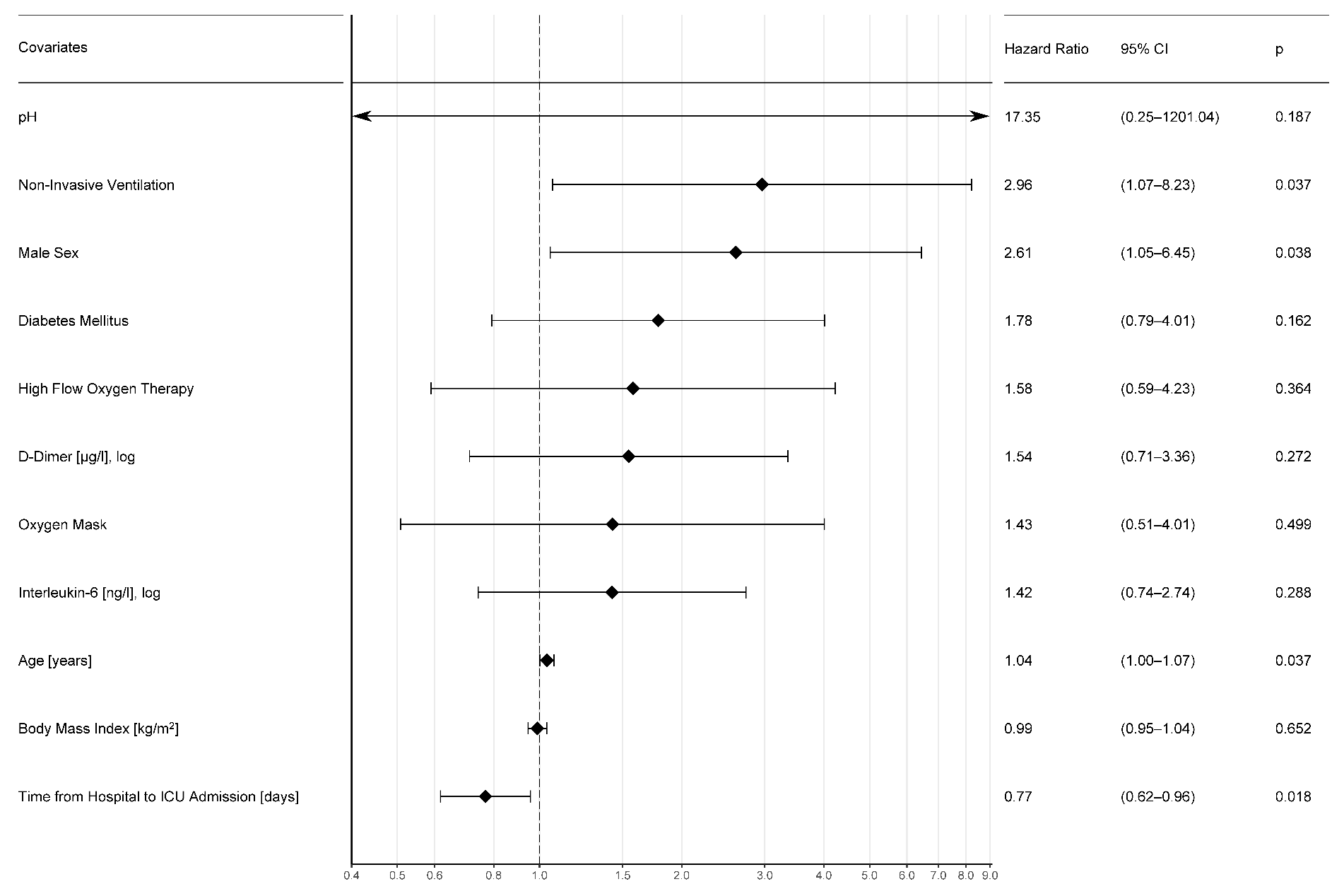
**e-Figure 8:** Multivariable adjusted COX regression model for ICU discharge (intubated patients only).

**
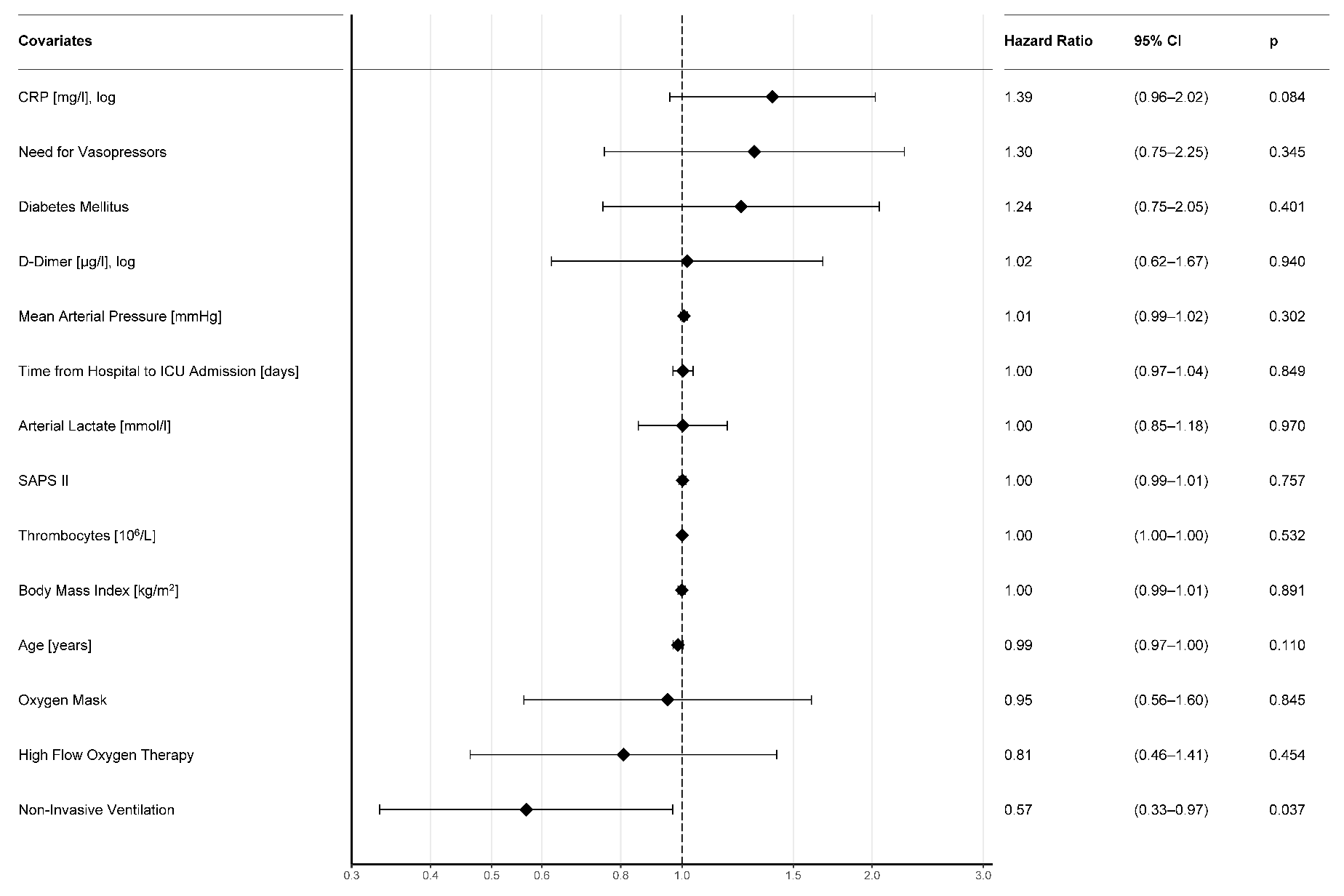
**

**e-Table 6:** Prognostic Model for the identification of patients with lower ICU mortality risk after a failed HFNC or NIV trial.

|  | **Estimate** | **Std. Error** | **z-value** | **p-value** |
| --- | --- | --- | --- | --- |
| **Intercept** | -6.10989 | 1.99125 | -3.068 | 0.00215 |
| **Age, years** | 0.04787 | 0.02339 | 2.046 | 0.04072 |
| **Respiratory Rate, 1/min** | 0.06557 | 0.03253 | 2.016 | 0.04382 |
| **Diabetes Mellitus** | 1.26969 | 0.52757 | 2.407 | 0.01610 |

**Cut-Off AUROC:** 134 pts

**Positive Likelihood Ratio (134 pts):** 2.4

**Negative Likelihood Ratio (134 pts):** 0.4


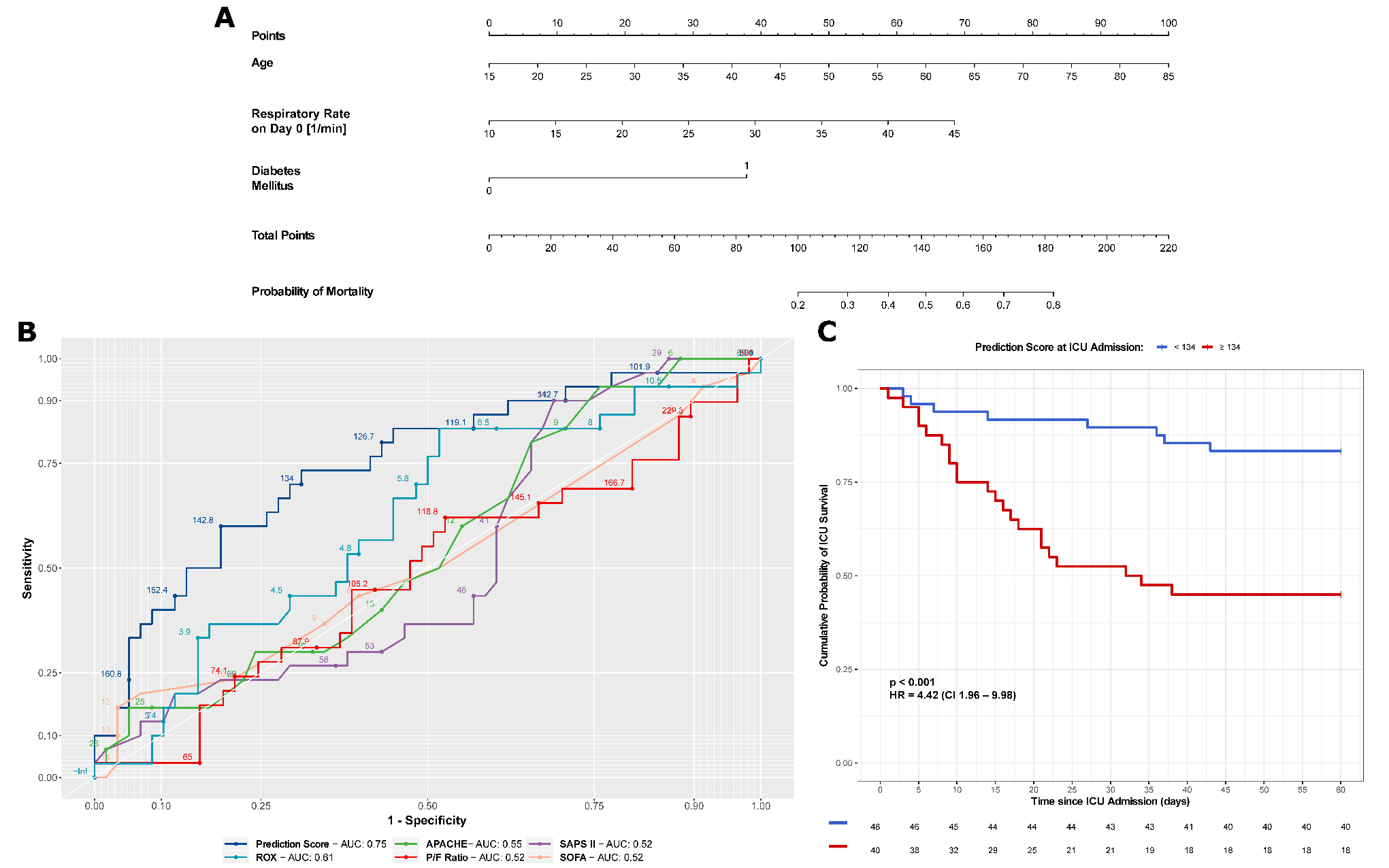
**e-Figure 9:** Nomogram, Receiver Operating Curves and stratified Kaplan Meier curve for a prognostic model identifying patients with lower ICU mortality risk after a failed HFNC or NIV trial. (A) Prediction score nomogram for intensive care unit mortality in high flow oxygen therapy and non-invasive positive-pressure ventilated patients progressing towards intubation and IMV, (B) receiver operating characteristic (ROC) curves for intensive care unit mortality stratified by severity scores on Day 0 and (C) Kaplan Meier Curve depicting ICU Mortality stratified by a prediction score of 134. APACHE II - Acute Physiology and Chronic Health Evaluation II; P/F Ratio - Partial Pressure of Arterial O_2_/ Fraction of Inspired O_2_; SAPS II - Simplified Acute Physiology Score II; SOFA - Sequential Organ Failure Assessment; ROX Index – SpO_2_/ FiO_2_/ Respiratory Rate; ICU – Intensive Care Unit.

**e-Table 7:** Area Under the Receiver Operating Curves (AUROCs) for the Prognostic Score versus classic severity scores. AUROC - Area Under the Receiver Operating Cure; CI - Confidence Interval; SOFA - Sequential Organ Failure Assessment; SAPS II - Simplified Acute Physiology Score II; APACHE II - Acute Physiology And Chronic Health Evaluation II; ROX Index – SpO2/ FiO2/ Respiratory Rate; PaO_2_/ FiO_2_ ratio - partial pressure of arterial O_2_/ fraction of inspired O_2_.

|  | **AUROC** | **95% CI** | **p vs. Score** |
| --- | --- | --- | --- |
| **Score** | 0.75 | 0.63 – 0.85 |  |
| **SOFA** | 0.52 | 0.36 – 0.61 | 0.002 |
| **SAPS II** | 0.52 | 0.35 – 0.61 | 0.003 |
| **APACHE II** | 0.55 | 0.33 – 0.57 | <0.001 |
| **ROX** | 0.61 | 0.48 – 0.74 | 0.044 |
| **PaO_2_/FiO_2_ Ratio** | 0.52 | 0.36 – 0.61 | 0.002 |

**References**

1. The ADTF. Acute Respiratory Distress Syndrome: The Berlin Definition. *JAMA.* 2012;307(23):2526-2533.

2. Group KW. KDIGO clinical practice guideline for acute kidney injury. 2012;2(1):1-138.

3. Thygesen K, Alpert JS, Jaffe AS, et al. Fourth universal definition of myocardial infarction (2018). *European Heart Journal.* 2019;40(3):237-269.

4. van Buuren S. Multiple imputation of discrete and continuous data by fully conditional specification. *Statistical Methods in Medical Research.* 2007;16(3):219-242.

5. Potthoff RF, Tudor GE, Pieper KS, Hasselblad V. Can one assess whether missing data are missing at random in medical studies? *Statistical Methods in Medical Research.* 2006;15(3):213-234.

6. Normand S-LT, Landrum MB, Guadagnoli E, et al. Validating recommendations for coronary angiography following acute myocardial infarction in the elderly: A matched analysis using propensity scores. *Journal of Clinical Epidemiology.* 2001;54(4):387-398.

7. Rubin DB. Using Propensity Scores to Help Design Observational Studies: Application to the Tobacco Litigation. *Health Services and Outcomes Research Methodology.* 2001;2(3):169-188.
